# The association between SARS-CoV-2 infection and preterm delivery: a prospective study with a multivariable analysis

**DOI:** 10.1186/s12884-021-03742-4

**Published:** 2021-04-01

**Authors:** Oscar Martinez-Perez, Pilar Prats Rodriguez, Marta Muner Hernandez, Maria Begoña Encinas Pardilla, Noelia Perez Perez, Maria Rosa Vila Hernandez, Ana Villalba Yarza, Olga Nieto Velasco, Pablo Guillermo Del Barrio Fernandez, Laura Forcen Acebal, Carmen Maria Orizales Lago, Alicia Martinez Varea, Begoña Muñoz Abellana, Maria Suarez Arana, Laura Fuentes Ricoy, Clara Martinez Diago, Maria Jesus Janeiro Freire, Macarena Alférez Alvarez-Mallo, Cristina Casanova Pedraz, Onofre Alomar Mateu, Cristina Lesmes Heredia, Juan Carlos Wizner de Alva, Rut Bernardo Vega, Montserrat Macia Badia, Cristina Alvarez Colomo, Antonio Sanchez Muñoz, Laia Pratcorona Alicart, Ruben Alonso Saiz, Monica Lopez Rodriguez, Maria del Carmen Barbancho Lopez, Marta Ruth Meca Casbas, Oscar Vaquerizo Ruiz, Eva Moran Antolin, Maria Jose Nuñez Valera, Camino Fernandez Fernandez, Albert Tubau Navarra, Alejandra Maria Cano Garcia, Carmen Baena Luque, Susana Soldevilla Perez, Irene Gastaca Abasolo, Jose Adanez Garcia, Maria Teulon Gonzalez, Alberto Puertas Prieto, Rosa Ostos Serna, Maria del Pilar Guadix Martin, Monica Catalina Coello, Elena Ferriols Perez, Africa Caño Aguilar, Maria Luisa De la Cruz Conty, Jose Antonio Sainz Bueno, Spanish Obstetric Emergency Group

**Affiliations:** 1 Department Gynaecology and Obstetrics, Puerta de Hierro University Hospital of Majadahonda, Majadahonda, Madrid, Spain; 2 Department Reproductive Medicine and Gynaecology, Obstetrics, Dexeus University Hospital, Barcelona, Catalunya Spain; 3 Department Gynaecology and Obstetrics, La Paz University Hospital. Madrid, Madrid, Spain; 4 Department Gynaecology and Obstetrics, San Carlos University Hospital. Madrid, Madrid, Spain; 5 Department Gynaecology and Obstetrics, Santa Caterina Hospital, Girona, Catalunya Spain; 6 Department Gynaecology and Obstetrics, University Hospital of Salamanca, Salamanca, Castilla y León Spain; 7 Department Obstetrics and Gynaecology, Quironsalud Madrid University Hospital. Madrid, Madrid, Spain; 8 Department Gynaecology and Obstetrics, Getafe University Hospital, Getafe, Madrid, Spain; 9 Department Gynaecology and Obstetrics, Doce de Octubre University Hospital, Comunidad de Madrid, Madrid, Spain; 10 Department Gynaecology and Obstetrics, Severo Ochoa University Hospital, Leganes, Madrid, Spain; 11 Department Gynaecology and Obstetrics, La Fe University and Polytechnic Hospital, Comunidad Valenciana, Valencia, Spain; 12 Department Obstetrics and Gynaecology, Sant Joan de Reus University Hospital, Reus, Catalunya Spain; 13 Department Gynaecology and Obstetrics, Regional University Hospital of Malaga, Malaga, Andalucía Spain; 14 Department Obstetrics and Gynaecology, University Hospital of Ferrol, Ferrol, A Coruña, Spain; 15 Department Gynaecology and Obstetrics, Doctor Josep Trueta University Hospital of Girona, Girona, Catalunya Spain; 16 Department Obstetrics and Gynaecology, University Hospital Complex of A Coruña, A Coruña, Spain; 17 Department Obstetrics and Gynaecology, HM Hospitals, Madrid, Spain; 18 Department Gynaecology and Obstetrics, University Hospital of Torrejon, Torrejon de Ardoz, Madrid, Spain; 19 Department Obstetrics and Gynaecology, Regional Hospital of Inca, Inca, Illes Balears Spain; 20 Department Obstetrics and Gynaecology, Parc Taulí Hospital, Barcelona, Catalunya Spain; 21 Department Gynaecology and Obstetrics, San Pedro de Alcántara Hospital, Caceres, Extremadura Spain; 22 Department Gynaecology and Obstetrics, Rio Hortega University Hospital, Valladolid, Castilla y León Spain; 23 Department Gynaecology and Obstetrics, Arnau de Vilanova University Hospital, Lleida, Catalunya Spain; 24 Department Gynaecology and Obstetrics, Valladolid University Hospital, Valladolid, Castilla y León Spain; 25 Department Gynaecology and Obstetrics, General University Hospital of Ciudad Real, Ciudad Real, Castilla-La Mancha Spain; 26 Department Obstetrics and Gynaecology, Germans Trias i Pujol University Hospital, Barcelona, Catalunya Spain; 27 Department Gynaecology and Obstetrics, University Hospital of Burgos, Burgos, Castilla y León Spain; 28 Department Obstetrics and Gynaecology, Joan XXIII University Hospital of Tarragona, Tarragona, Catalunya Spain; 29 Department Gynaecology and Obstetrics, Infanta Sofia University Hospital, San Sebastian de los Reyes, Madrid, Spain; 30 Department Obstetrics and Gynaecology, Poniente Hospital of Almería, El Ejido, Andalucía Spain; 31 Department Obstetrics and Gynaecology, University Hospital of Cabueñes, Gijon, Asturias Spain; 32 Department Obstetrics and Gynaecology, Son Espases University Hospital, Palma de Mallorca, Illes Balears Spain; 33 Department Obstetrics and Gynaecology, Virgen de la Luz Hospital, Cuenca, Castilla-La Mancha Spain; 34 Department Gynaecology and Obstetrics, University Assistance Complex of Leon, Leon, Castilla y Leon Spain; 35 Department Obstetrics and Gynaecology, Son Llatzer University Hospital, Palma de Mallorca, Illes Balears Spain; 36 Department Gynaecology and Obstetrics, University Hospital of El Tajo, Aranjuez, Madrid, Spain; 37 Department Gynaecology and Obstetrics, Infanta Margarita Hospital, Andalucía, Cabra, Spain; 38 Department Obstetrics and Gynaecology, University Hospital of Jerez de la Frontera, Jerez de la Frontera, Andalucía Spain; 39 Department Obstetrics and Gynaecology, Txagorritxu University Hospital of Araba, Vitoria-Gasteiz, País Vasco Spain; 40 Department Obstetrics and Gynaecology, Central University Hospital of Asturias, Oviedo, Asturias Spain; 41 Department Gynaecology and Obstetrics, University Hospital of Fuenlabrada, Fuenlabrada, Madrid, Spain; 42 Department Obstetrics and Gynaecology, Virgen de las Nieves University Hospital, Granada, Andalucía Spain; 43 Department Gynaecology and Obstetrics, Virgen de Valme University Hospital, Sevilla, Andalucía Spain; 44 Department Obstetrics and Gynaecology, Virgen Macarena University Hospital, Sevilla, Andalucía Spain; 45 Department Gynaecology and Obstetrics, Virgen de la Concha Hospital, Zamora, Castilla y León Spain; 46 Department Gynaecology and Obstetrics, Parc de Salut Mar University Hospital, Barcelona, Catalunya Spain; 47 Department Obstetrics and Gynaecology, San Cecilio University Hospital of Granada, Granada, Andalucía Spain; 48 Fundación de Investigación Biomédica, Puerta de Hierro University Hospital of Majadahonda, Majadahonda, Madrid, Spain; 49 Department Gynaecology and Obstetrics, University of Seville, G. Chacon (Viamed Santa Angela de la Cruz Hospital), Sevilla, Andalucia Spain

**Keywords:** SARS-CoV-2, Coronavirus, COVID-19, Pregnancy, Premature birth, Premature rupture of membranes, Intensive care units, neonatal

## Abstract

**Background:**

To determine whether severe acute respiratory syndrome coronavirus 2 (SARS-CoV-2, the cause of COVID-19 disease) exposure in pregnancy, compared to non-exposure, is associated with infection-related obstetric morbidity.

**Methods:**

We conducted a multicentre prospective study in pregnancy based on a universal antenatal screening program for SARS-CoV-2 infection. Throughout Spain 45 hospitals tested all women at admission on delivery ward using polymerase-chain-reaction (PCR) for COVID-19 since late March 2020. The cohort of positive mothers and the concurrent sample of negative mothers was followed up until 6-weeks post-partum. Multivariable logistic regression analysis, adjusting for known confounding variables, determined the adjusted odds ratio (aOR) with 95% confidence intervals (95% CI) of the association of SARS-CoV-2 infection and obstetric outcomes. Main outcome measures: Preterm delivery (primary), premature rupture of membranes and neonatal intensive care unit admissions.

**Results:**

Among 1009 screened pregnancies, 246 were SARS-CoV-2 positive. Compared to negative mothers (763 cases), SARS-CoV-2 infection increased the odds of preterm birth (34 vs 51, 13.8% vs 6.7%, aOR 2.12, 95% CI 1.32–3.36, *p* = 0.002); iatrogenic preterm delivery was more frequent in infected women (4.9% vs 1.3%, *p* = 0.001), while the occurrence of spontaneous preterm deliveries was statistically similar (6.1% vs 4.7%). An increased risk of premature rupture of membranes at term (39 vs 75, 15.8% vs 9.8%, aOR 1.70, 95% CI 1.11–2.57, *p* = 0.013) and neonatal intensive care unit admissions (23 vs 18, 9.3% vs 2.4%, aOR 4.62, 95% CI 2.43–8.94, *p* <  0.001) was also observed in positive mothers.

**Conclusion:**

This prospective multicentre study demonstrated that pregnant women infected with SARS-CoV-2 have more infection-related obstetric morbidity. This hypothesis merits evaluation of a causal association in further research.

**Supplementary Information:**

The online version contains supplementary material available at 10.1186/s12884-021-03742-4.

## Key message

This prospective multicentre study revealed that pregnant women infected with SARS-CoV-2 have more infection-related obstetric morbidity (Preterm birth, premature rupture of membranes at term and neonatal intensive care unit admissions).

## Background

Severe acute respiratory syndrome coronavirus 2 (SARS-CoV-2), identified in December 2019, is the cause of the illness named COVID-19 [1, 2]. With more than 249,000 confirmed cases and more than 28,700 deaths by 20th August 2020, Spain remains one of the European countries most severely affected by the ongoing COVID-19 pandemic [3, 4]. Spain also established a universal screening programme for pregnancies in light of the higher disease exposure. We observed that obstetric intervention may influence the clinical course of the disease [5–7]. The cohort of pregnant women assembled through this programme lends itself to evaluation of concerns about obstetric outcomes.

The majority of non-pregnant patients with SARS-CoV-2 infection have uncomplicated or mild illness (81%), some will develop severe illness associated with cytokine-mediated inflammation phenomena such as IL-6 associated with the need for mechanical ventilation [8]. Initial studies have reported similar involvement in pregnant patients [9]. The inflammatory mediators associated SARS-CoV-2 infection have previously been related to poor perinatal outcomes [10, 11]. This background naturally leads to the question as to whether SARS-CoV-2 infection affects pregnancy adversely.

We hypothesised that SARS-CoV-2 infection in pregnancy, compared to non-infection, would increase infection-related obstetric morbidity including preterm birth and premature rupture of membranes which in turn would increase the admissions of the neonate to intensive care units. We tested the hypothesis in a multivariable logistic regression analysis, adjusting for the effect of known confounding variables.

## Methods

This was a multicentre prospective study of consecutive cases of SARS-CoV-2 infection in a pregnancy cohort registered by the Spanish Obstetric Emergency group in 45 hospitals [12]. The registry’s objective updates were approved by the coordinating hospital’s Medical Ethics Committee on March 23rd, 2020 (reference number: PI 55/20); each collaborating center subsequently obtained protocol approval locally. The registry protocol is available in ClinicalTrials.gov, identifier: NCT04558996, and in Additional File 1. A complete list of the centers contributing to the study is provided in Supplementary Table 1, Additional File 2. Upon recruitment, given the contagiousness of the disease and the lack of personal protection equipment, mothers consented by either signing a document (Additional File 3), when possible, or by giving permission verbally which was recorded in the patient’s chart. A specific database was designed for recording information regarding SARS-CoV-2 infection in pregnancy. Data were entered by the lead researcher for each center after delivery, with a follow-up of 6-weeks postpartum in order to detect complications or symptomatic infections. We developed an analysis plan using recommended contemporaneous methods and followed existing guidelines for reporting our results (Supplementary Table 2, Additional File 2) [13].

### Infected group

**Fig. 1:**
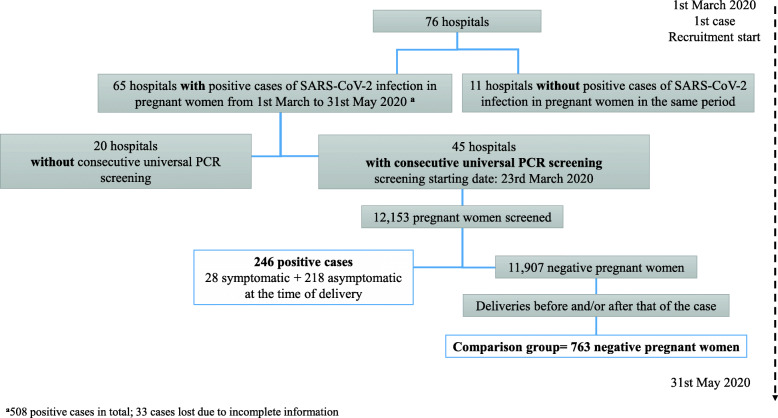
Study Flow chart

We included infected obstetric patients detected by routine screening for SARS-CoV-2 infection, which was carried out in every pregnant at admission on delivery ward during the study period from the 23rd of March to the 31st of May 2020 (Fig. 1). SARS-CoV-2 infection was diagnosed by positive double-sampling polymerase-chain-reaction (PCR) from nasopharyngeal swabs. All identified cases were included in the study, irrespective of clinical signs and symptoms or the result of another serological test. In those cases, with a clinical presentation of SARS-CoV-2 infection, it was classified following the WHO division for adults: mild symptoms, mild-moderate pneumonia, severe pneumonia and septic shock [14].

### Non-infected comparison group

Non-infected patients were those defined by a negative PCR in the routine screening carried out in every pregnant at admission on delivery ward. Among these PCR negative mothers, each center identified 2–3 pregnancies delivered immediately before and/or after delivery of each SARS-CoV-2 infected mother (Fig. 1). This method of identifying mothers not exposed to SARS-CoV-2 infection was deployed to adjusted for center conditions at the time of delivery and decreased the risk of bias.

#### Study information

Hospitals collected the encoded information in two separate phases: during the enrolment period that occurred at the time of the SARS-CoV-2 test during pregnancy and within 6 weeks after birth. Information regarding the demographic characteristics of each pregnant woman, comorbidities and current obstetric history was extracted from the clinical history and from the interview with the patient; subsequently, age and race were categorized following the classification used by the CDC [15]. Definitions of obstetric conditions followed international criteria [16–18]. Perinatal events, medical and obstetric complications were recorded. Preterm deliveries were classified as spontaneous onset (including those resulting from a PPROM), induced labour/C-section due to PPROM and iatrogenic (not associated with PPROM). Patients were followed until six weeks postpartum. Neonatal events were recorded until 14 days postpartum. Recorded variables are listed in Supplementary Table 1, Additional File 1. A total of 33 dropout cases were recorded from the beginning of the registry; the reasons were incomplete information in the registry database, did not participate in the six-week postpartum follow-up and/or voluntary withdrawal of the patient.

#### Data analysis

We estimated that a sample size of 246 SARS-CoV-2 positive pregnancies with a ratio 1:3 of negative pregnancies would detect a 6% difference in proportions between groups with a power of 80% at a significance level of 5%, assuming a 7% event rate of preterm deliveries in the non-infected group [19]. This level of event rate also permitted us to build logistic regression models without overfitting using the 10:1 event per variable rule.

For the descriptive analysis of the data, absolute and relative frequencies were used in the case of categorical variables and means and ranges in the case of quantitative variables. The possible association of both the characteristics of the patients and the outcomes collected with SARS-CoV-2 infection was analysed using the Pearson’s Chi-square test or Fisher’s exact test and the Mann-Whitney U test (after checking the absence of normality of the data using the Kolmogorov-Smirnov test). Statistical tests were two-sided and were performed with SPSS V.20 (IBM Inc., Chicago, Il, USA); statistically significant associations were considered to exist when the *p* value was less than 0.05.

For computing measures of association of the outcomes of interest that were statistically significant in the univariate analysis (and with enough number of events) with SARS-CoV-2 infection, the influence of known and suspected measured confounding factors was controlled for multivariable logistic regression modelling in order to derive adjusted odds ratios (aOR) with 95% confidence intervals (95% CI). Models were built for each outcome separately, incorporating a range of independent variables appropriate for the adjustment of the association between SARS-CoV-2 infection and that outcome. The selection process for variables was driven by causal knowledge for the adjustment of confounding, based on previous findings and clinical constraints [11, 14–18]. Besides SARS-CoV-2 positivity, the preterm delivery model included Ethnicity [categorized as white European, Latin American and other ethnic groups (black non-Hispanic, Asian non-Hispanic and Arab)], multiple pregnancy, in vitro fertilization, gestational hypertensive disorders (moderate or severe preeclampsia and HELLP), miscarriage risk and clinical and ultrasound prematurity screening; the spontaneous preterm delivery model included ethnicity (categorized as above), multiple pregnancy, miscarriage risk and clinical and ultrasound prematurity screening; the premature rupture of membranes at term (PROM) model included multiple pregnancy, miscarriage risk, cough, obesity (BMI > 30 kg/m^2^) and smoking [categorized as smokers (actual and ex-smokers) and non-smokers]; the preterm premature rupture of membranes (PPROM) model included multiple pregnancy and miscarriage risk; and the neonate intensive care unit (NICU) admission model included multiple pregnancy, gestational hypertensive disorders and clinical and ultrasound prematurity screening as independent variables.

A complete list of the final set of covariates is provided with each model in the results section. The modelling was conducted after excluding cases with missing data. A prematurity screening program was not established in all participating hospitals and that variable had 11.3% of missing values, whereas the remaining variables had less than 1.2% of missing values. Regression analyses were carried out using lme4 package in R, version 3.4 (RCoreTeam, 2017) [20].

## Results

One thousand and nine (1009) patients were analysed. 246 pregnant women in the infected group and 763 in the non-infected group. Of the 246 positive cases, 88.6% (*n* = 218) were asymptomatic at delivery while 11.4% (*n* = 28) were symptomatic. Of the asymptomatic women, 44 (20.2%) had previously presented symptoms and 174 (79.8%) were totally asymptomatic. On the other hand, of the pregnant women who showed symptoms at the time of delivery, 24 (85.7%) cases corresponded to mild symptoms (being the most prevalent, cough 33.3%, and anosmia 20.8%, followed by fatigue/discomfort, fever and dyspnoea), 2 (7.1%) pregnant women presented mild-moderate pneumonia and another 2 (7.1%) pregnant women had developed severe pneumonia. No case of septic shock or maternal death was recorded in pregnant women with SARS-CoV-2 infection included in the study.

The demographic characteristics, comorbidities and current obstetric history of the positive cohort and the subsample of negative patients concurrent in time deliveries (246 vs 763) are shown in Table 1. The only variable in which statistically significant differences were observed was ethnicity, being significantly higher the proportion of Latin American women in the infected cohort compared to the non-infected group (*p* <  0.001; OR = 2.85, 95% CI: 1.96–4.15), while the opposite was true for White European patients (*p* < 0.001, OR = 0.49, 95% CI: 0.36–0.67).

**Table 1:** Demographic characteristics, comorbidities and current obstetric history of the study participants (*n* = 1009)

	Infected Group	Non-Infected Group	***p***-value	
Number	246	763		
**Maternal characteristics**				
Maternal age (years; mean/range)	32.6 (18–45)	32.5 (18–49)	0.671	
Age Range				
18–24	28 (11.5%)	70 (9.2%)	0.309	
25–34	114 (46.3%)	400 (52.8%)	0.097	
35–49	101 (41.1%)	287 (37.9%)	0.335	
Ethnicity				
White European	158 (64.2%)	600 (78.9%)	< 0.001*	0.49 (0.36–0.67)
Latino Americans	59 (24.0%)	76 (10.0%)	< 0.001*	2.85 (1.96–4.15)
Black non-Hispanic	8 (3.3%)	11 (1.4%)	0.100	
Asian non-Hispanic	4 (1.6%)	18 (2.4%)	0.494	
Arab	17 (6.9%)	55 (7.2%)	0.875	
Nulliparous	82 (38.5%)	254 (37.4%)	0.775	
Smoking ^a^	35 (14.2%)	94 (12.3%)	0.436	
**Maternal comorbidities**				
Obesity (BMI > 30 kg/m^2^)	33 (13.7%)	119 (16.8%)	0.255	
Cardiovascular comorbidities				
Chronic Heart Failure ^b^	3 (1.2%)	7 (1.0%)	0.744	
Pre-pregnancy HBP	3 (1.2%)	6 (0.9%)	0.587	
Pulmonary comorbidities				
Chronic Pulmonary Disease (not asthma)	1 (0.4%)	1 (0.1%)	0.445	
Asthma	4 (1.7%)	25 (3.5%)	0.144	
Hematologic comorbidities				
Chronic Blood Disease	1 (0.4%)	4 (0.6%)	1.000	
Thrombophilia	3 (1.2%)	13 (1.8%)	0.546	
Antiphospholipid Syndrome	0 (0.0%)	1 (0.1%)	1.000	
Chronic kidney disease	0 (0.0%)	2 (0.3%)	1.000	
Chronic liver disease	3 (1.2%)	2 (0.3%)	0.097	
Rheumatic disease	1 (0.4%)	7 (1.0%)	0.397	
Diabetes mellitus	1 (0.4%)	10 (1.5%)	0.305	
Depressive syndrome	2 (0.8%)	8 (1.1%)	0.680	
**Current obstetric history**				
Gestational age at enrolment (weeks+days; mean/range)	38 + 1 (27–42)	38 + 6 (21–42)	< 0.001	
Multiple pregnancy	6 (2.4%)	31 (4.1%)	0.239	
In Vitro Fertilization	15 (6.1%)	27 (3.5%)	0.081	
Haemoglobin < 10 g/dL	6 (2.5%)	39 (5.4%)	0.065	
Platelets < 100,000/μL	3 (1.2%)	2 (0.3%)	0.104	
Pregnancy-induced hypertension ^c^	12 (4.9%)	34 (4.8%)	0.962	
Gestational diabetes	17 (7.0%)	61 (8.4%)	0.476	
Intrauterine growth restriction	13 (5.3%)	27 (3.8%)	0.303	
High Risk Preeclampsia Screening	8 (3.7%)	38 (5.8%)	0.230	
High-risk Chromosomal Abnormality Screening	4 (1.7%)	18 (2.6%)	0.428	
Clinical and Ultrasound Prematurity Screening	5 (2.3%)	21 (3.3%)	0.461	
Miscarriage risk	11 (4.5%)	17 (2.2%)	0.062	

Data are shown as n (% of total), except where otherwise indicated

OR: odds ratio; CI: confidence interval; BMI: Body Mass Index; HBP: High Blood Pressure

*Statistically significant differences: OR and 95% CI were estimated

^a^ Current smoker and ex-smoker

^b^ Including Congenital Heart Disease, not Hypertension

^c^ Hypertension + preeclampsia

When the possible association of perinatal and neonatal events with SARS-CoV-2 infection was analysed by univariate and multivariable logistic regression using complete case analyses (without imputation for missing values) (Tables 2 and 3), twice as many deliveries with less than 37 weeks of gestation were observed in the infected cohort (13.8%) than in the negative group (6.7%) (*p* = 0.002), with an adjusted OR equal to 2.12 (95% CI: 1.32–3.36), although no statistically significant differences had been observed in the clinical and ultrasound screening for prematurity between both groups (*p* = 0.461) (Table 1). Among preterm deliveries, iatrogenic preterm delivery (not associated with PPROM) was practically four times more frequent in infected by SARS-CoV-2 pregnant women than in the non-infected group (4.9% vs 1.3%, *p* = 0.001), while the occurrence of spontaneous preterm deliveries was not affected by SARS-CoV-2 infection status (*p* = 0.760, adjusted OR = 1.10, 95% CI: 0.57–2.06) (Table 3). In the positive group, symptomatic SARS-CoV-2 infection were present in 5 (42%) out of 12 iatrogenic preterm deliveries, while this was the case in only 3 (20%) out of 15 spontaneous preterm deliveries.

**Table 2:** Maternal and neonatal outcomes of the study participants (n = 1009)

	Infected Group	Non-Infected Group	p-value
Number	246	763	
**Perinatal outcome**			
Gestational age at delivery (weeks+days; mean/range)	38 + 4 (27–42)	39 + 0 (23–42)	**0.010**
Type of delivery			
Cesarean	55 (22.4%)	143 (18.7%)	0.214
Eutocic	170 (69.1%)	506 (66.3%)	0.419
Instrumental	21 (8.5%)	114 (14.9%)	**0.010**
Preterm deliveries (< 37 weeks of gestational age)	34 (13.8%)	51 (6.7%)	**0.001**
Spontaneous delivery (including PPROM)	15 (6.1%)	36 (4.7%)	0.390
Induced/Elective C-section due to PPROM	7 (2.8%)	5 (0.7%)	**0.012**
Iatrogenic delivery (no PPROM)	12 (4.9%)	10 (1.3%)	**0.001**
Causes of preterm iatrogenic delivery:			
COVID-19 mild symptoms	3/12	0/10	
Pneumonia	3/12	0/10	
Severe preeclampsia	4/12	1/10	0.323
PROM	39 (15.8%)	75 (9.8%)	**0.009**
PPROM	11 (4.5%)	15 (2.0%)	**0.031**
Gestational age at PPROM (weeks+days; mean/range)	33 + 5 (28–36)	33 + 6 (28–36)	0.610
***Medical and obstetrical complications***			
Admitted in ICU	5 (2.0%)	2 (0.3%)	**0.011**
Days in ICU (mean/range)	9.5 (6–14)	2 (2–2)	
***Obstetrical complications***			
Hemorrhagic events	10 (4.1%)	34 (4.5%)	0.794
Abruptio placentae	2 (0.8%)	1 (0.1%)	0.149
Postpartum hemorrhage	8 (3.3%)	33 (4.3%)	0.459
Gestational hypertensive disorders	11 (4.5%)	44 (5.8%)	0.436
Severe preeclampsia	6 (2.4%)	3 (0.4%)	**0.008**
Admitted in ICU	2/6	0/3	
Invasive ventilation	0/6	0/7	
Moderate preeclampsia	5 (2.0%)	41 (5.4%)	**0.025**
**Neonatal data**			
Apgar 5 score < 7	5 (2.0%)	8 (1.1%)	0.325
Umbilical artery pH < 7.10	6 (3.0%)	24 (3.8%)	0.608
Admitted in NICU	23 (9.3%)	18 (2.4%)	**0.001**
Days in NICU (mean/range)	13.8 (1–48)	10.7 (2–26)	0.379
Cause of NICU admission:			
Prematurity	15/23	12/18	
Respiratory distress	2/23	6/18	
Neonatal COVID-19 PCR testing within the first 48 h	196 (79.7%)	0 (0.0%)	
***Stillbirth***	3 (1.2%)	1 (0.1%)	**0.047**
***Neonatal mortality***	0 (0.0%)	1 (0.1%) ^a^	1.000
**6 weeks mother follow-up**			
Mastitis	1 (0.4%)	1 (0.1%)	0.428
**14 days neonate follow-up**			
Readmission due to COVID-19	0 (0.0%)	0 (0.0%)	

Data are shown as n (% of total), except where otherwise indicated

COVID-19: Coronavirus disease 2019; PROM: Premature rupture of membranes; PPROM: Preterm Premature Rupture of Membranes; ICU: Intensive care unit; NICU: Neonatal intensive care unit

^a^ Prematurity causes, gestational age at delivery was 24 weeks

**Table 3:** Odds ratio and adjusted odds ratio for obstetric outcomes associated with SARS-CoV-2 exposure in pregnancy

		Outcomes			
	Preterm delivery	Spontaneous preterm delivery	PROM	PPROM	NICU admission
**Univariate analysis (OR)**					
SARS-CoV-2 positive	2.23	1.31	1.72	2.33	4.27
95% CI	1.41–3.54	0.69–2.39	1.14–2.62	1.06–5.15	2.26–8.05
p-value	0.001	0.390	0.009	0.031	0.001
**Multivariate analysis (aOR)**					
SARS-CoV-2 positive	2.12	1.10	1.70	2.26	4.62
95% CI	1.32–3.36	0.57–2.06	1.11–2.57	0.99–4.98	2.43–8.94
*p*-value	0.002	0.760	0.013	0.045	< 0.001
In Vitro Fertilization	2.37	–	–	–	–
95% CI	0.97–5.16	–	–	–	–
p-value	0.041	–	–	–	–
Miscarriage Risk	2.61	4.19	–	2.69	–
95% CI	0.92–6.42	1.35–10.88	–	0.42–9.91	–
p-value	0.050	0.006	–	0.198	–
Ethnicity					
Latin American vs White European	–	2.11 (1.04–4.09)	–	–	–
Other Ethnic Groups vs White European	–	0.38 (0.06–1.27)	–	–	–
p-value	–	0.031 and 0.188	–	–	–
Multiple pregnancy	–	–	1.86*10^−7^	–	3.72
95% CI	–	–	0.00 – .	–	1.02–10.73
p-value	–	–	0.981	–	0.025
Gestational Hypertensive Disorders	–	–	–	–	3.63
95% CI	–	–	–	–	1.28–8.91
p-value	–	–	–	–	0.008

*OR* Odds Ratio

*aOR* adjusted Odds Ratio

*95% CI* 95% Confidence Interval

*PROM* Premature Rupture of Membranes at term

*PPROM* Preterm Premature Rupture of Membranes

*NICU* Neonatal Intensive Care Unit

Multivariable logistic regression used for each outcome as dependent variable and COVID-19 exposure in pregnancy and known/suspected confounding variables as independent variables (see Methods for details)

-- Variables not included or not held in the multivariate model

Similarly, a higher risk of premature rupture of membranes, at term (PROM) and preterm (PPROM), was observed in the infected group (*p* = 0.009 and *p* = 0.031, respectively) (Table 2). In the case of PROM, the finding of multivariable logistic regression was consistent with the above result, with a 70% increase of occurrence in infected patients compared to non-infected (adjusted OR = 1.70, 95% CI: 1.11–2.57) (Table 3).

No maternal deaths were recorded in the 1009 patients in the study, but there were intrauterine fetal deaths, with the proportion of these being considerably higher in patients in the positive group than in the subsample of negative patients (1.2% vs 0.1%, *p* = 0.047) (Table 2).

When the information regarding the neonate was analysed (Tables 2 and 3), those born to mothers with SARS-CoV-2 infection were admitted to the NICU significantly more often than those born to non-infected mothers (*p* < 0.001, adjusted OR = 4.62, 95% CI: 2.43–8.94). Prematurity and respiratory distress were the main causes of NICU admission (Table 2), while none of these admissions were due to SARS-CoV-2 infection in newborns. In 189 (76.8%) of the SARS-CoV-2 infection cases, a PCR analysis was performed on nasopharyngeal and/or oropharyngeal samples of the newborns; 147 were performed during the first 12 h of life, three of which were positive, and another 42 were performed until 48 h of life, all resulting negative. The 3-initial positive newborns were retested at 48 h, with final negative results.

## Discussion

### Main findings

Through a multicentre prospective study, we analysed the relationship between SARS-CoV-2 exposure and infection-related obstetric outcomes. We found, using multivariable models adjusting for confounding factors, that the pregnant women with SARS-CoV-2 infection had more preterm births, premature rupture of membranes at term and NICU admissions compared to the pregnant woman who were not exposed.

### Strengths and weaknesses

Ours is a study with a group of positive mothers carried out during a difficult pandemic situation whose continuing objective is to investigate the influence of SARS-CoV-2 infection on delivery and the puerperium. We wish to obtain the best epidemiological information in the shortest possible time with a follow-up 6 weeks after delivery. Patient recruitment continues in our registry and this is an initial analysis. Our work is one of the first multicentre prospective studies to analyse the relationship between SARS-CoV-2 infection and prematurity. The relationship that we establish with premature rupture of membranes raises future lines of research.

The most important limitation of our work is the inability to compare infected patients with uninfected patients from the beginning due to the lack of diagnostic tests and the health sector crisis that occurred. When a screening system was established, there were not as many patients with severe symptoms and the number of events reduced the ability to analyse some effects of symptomatic SARS-CoV-2 infection. Many cases of obstetric severe preeclampsia, haemorrhage, pulmonary thromboembolism and abruptio occurred mainly in the months of March and April before many centres started screening programmes and the cohort study began, so no distinction has been made between the different clinical presentations of the disease. We could not do a multivariable analysis of such conditions.

No serological test was performed on patients who had a negative PCR test, either because the tests were not available at the time of recruitment or because they did not have a proven sensitivity. In some cases, these patients may have already had the disease. No serology was performed during those months on asymptomatic PCR-positive patients to confirm their disease and immune response. Our study is best understood if the results are interpreted under this premise and therefore our group continues to recruit patients to seek more associations, explanations and causations. This work reflects the conditions of patients with SARS-CoV-2 infection at the time of delivery and the puerperium. It has not analysed the course of the disease during pregnancy, nor has it recorded late abortions, vertical transmission, or causes of intrauterine mortality.

In addition, we acknowledge as a limitation the absence of the complete cohort screened from analysis. In this sense our study has a hybrid design. The PCR negative comparison group was a subsample of the screen negative cohort from all 45 hospitals that had PCR positive mothers. The concurrent method applied for selection of non-infected cohort allowed for a comparison unaffected by difference in time of exposure and outcome assessment.

### Comparison with other studies

The symptoms of the patients in our study do not differ from those already published [21, 22]. Although most did not have any symptoms, we did find an increase in obstetric pathology in these patients, which in our opinion indicates that in the pregnant woman with asymptomatic SARS-CoV-2 infection there is a specific obstetric pathology that needs to be recognised. In the same way as other authors, we have also found a demographic factor, such as ethnicity, that increases the possibility that a patient has SARS-CoV-2 infection [15, 23]. It is necessary to know if there is a component of genetic susceptibility or if there are social factors that explain this association. There are already studies that relate this situation to less access to healthcare resources or the possibility of confinement which complies with healthcare measures [24].

Patients with SARS-CoV-2 infection are at increased risk of preterm delivery associated with increased iatrogenic preterm delivery. The explanation for this risk is the need to end the pregnancy due to maternal diseases, such as severe pre-eclampsia and pneumonia, which are more frequent in these patients and lead to more labour inductions. A unique and novel finding in our study is the association between premature rupture of membranes at term and SARS-CoV-2 infection. PROM may result in immediate risks and subsequent problems including maternal or neonatal infection [25]. One of the possible explanations we found for this association is the activation of a series of mediators and biochemical pathways of inflammation in the premature rupture of membranes and premature delivery that are also found in SARS-CoV-2 infection, such as macrophages or IL-6 [26]. The studies demonstrating the influence of IL-6 on preterm delivery are a strong basis for studying this association [27]. Cytokines are vital in regulating immunological and inflammatory responses. Among them, IL-6 is of major importance because there is evidence that circulating IL-6 levels are closely linked to the severity of the SARS-CoV-2 infection [28]. There are already treatments that are indicated based on these findings [29].

We observed a significant increase in the stillbirth rate in the univariate analysis alone. The role of inflammation mediators in these deaths could be the subject of a line of research because it is known that women without SARS-CoV-2 infection who have a pregnancy loss, have significantly higher amniotic fluid IL-6 concentration levels than those with a normal outcome [30]. We found no differences in mortality or early or late neonatal morbidity related to SARS-CoV-2 exposure in our study, unlike reports from other series [31, 32]. .There is a higher risk that the children of SARS-CoV-2 infection mothers enter the NICU, with prematurity being one of the determining factors. All newborns were followed for at least 14 days by the different neonatology units of the participating hospitals, without any case of neonatal SARS-CoV-2 infection being detected in that period.

To date, there has been indirect evidence on placental involvement which would explain our findings [33, 34]. Our results derived using multivariable analyses confirm those of the cases series published at the beginning of the pandemic that described preterm deliveries and premature rupture of the membranes [21, 31, 35].

## Conclusion

Pregnant SARS-CoV-2 infection patients are a population at risk of suffering preterm deliveries, and the disease has an impact on NICU admissions. Premature rupture of membranes at term and preterm are more frequent in patients with SARS-CoV-2 infection.

## Supplementary Information

**Additional file 1 **Registry Protocol. **Table S1.** List of variables in the Registry (Appendix 5 of Registry Protocol).

**Additional file 2: Table S1**. List of hospitals included in the study (*n* = 45); and **Table S2**. STROBE Statement—checklist of items that should be included in reports of observational studies.

**Additional file 3.** Patient Consent Form.

## Data Availability

The data that support the findings of this study are available from the Institute of Health-Carlos III and the Spanish Ministry of Health (registry protocol in ClinicalTrials.gov: NCT04558996) and has been previously published by Encinas Pardilla et al. [12]. Data are however available from the authors upon reasonable request and with permission of the Institute of Health-Carlos III and the Spanish Ministry of Health.

## Abbreviations

OR: Odds ratio
aOR: Adjusted odds ratio
NICU: Neonate intensive care unit
PCR: Polymerase-chain-reaction
PROM: Premature rupture of membranes at term
PPROM: Premature rupture of membranes at term
