# Supplementary material for: The association between SARS-CoV-2 infection and preterm delivery: a prospective study with a multivariable analysis
Source: BMC Pregnancy Childbirth. 2021 Apr 1;21:273. doi: 10.1186/s12884-021-03742-4 (PMC8016158; doi:10.1186/s12884-021-03742-4)
Supplement: Supplementary file 1 — Additional file 1 Registry Protocol. Table S1. List of variables in the Registry (Appendix 5 of Registry Protocol). [file 12884_2021_3742_MOESM1_ESM.zip › Additional File 1R4.pdf]

Additional File 1. Registry Protocol

Supplementary Table 1. List of variables in the Registry (Appendix 5 of Registry Protocol)

Table of Contents

Summary 3
- Study title 3
- Protocol number and version 3
- Sponsors 3
- Principal investigator of the registry 3
- Funding 3
- Abstract 3

Study rationale and background 4
- Clinical course of SARS-CoV2 infection in pregnant women 4
- Vertical transmission 5
- Rationale 5

Study hypothesis and objectives 5
- Hypothesis 5
- Objectives 5

Study design 6

Definitions 6
- Definition of case and healthy patient for the study 6
- Definitions of symptomatic and asymptomatic patient 6

Study population and selection criteria 7

For the characterization of the clinical course of SARS-CoV-2 infection during pregnancy, the pre-study sample size calculation has not been carried out, as registry-based studies generally tend to be comprehensive without involving hypotheses. 7
- Exclusion criteria 7

Overall sample size 7

Data sources and work plan 7
- Patient referral sources 7
- Data collection 7
- Data quality assurance plan 8
- Heterogeneity tests 8

EXTRACTION OF DATA FROM THE REGISTRY: SUBSTUDIES 9
- Substudy 1: Prospective observational study on delivery and vertical transmission to the new-born. 9
- Substudy 2: Prospective observational study on maternal and neonatal morbidity in pregnant women with COVID-19 11
- Substudy 3. Cohort study on obstetric pathology. 13

Substudy 4. Epidemiological prevalence study 15
Management and reporting of adverse reactions *17*
Ethical aspects *17*
Informed consent 17
Safety Measures and Confidentiality 18
Use of the data contained in the registry 18
References *18*

Summary

Study title

Spanish Registry of Pregnant Women with COVID-19

Protocol number and version

Number 55/20. Version V8.

Sponsors

This registry is a project promoted by Dr. Oscar Martínez Pérez of the Obstetrics and Gynaecology department of the Puerta de Hierro University Hospital.

Principal investigator of the registry

National coordinator: Dr. Óscar Martínez Pérez. Obstetrics and Gynaecology Department. Puerta de Hierro University Hospital. Majadahonda.

Epidemiologist: Maria Luisa de la Cruz Conti

Researchers for each site: 100 sites from 32 Spanish provinces are included (Appendix 1)

Funding

Neither the hospitals nor the participating investigators will receive any financial compensation for their collaboration. A bank account has been opened at the hospital's Biomedical Research Foundation to receive donations: COV20/00072 - SARS-COV-2 and the COVID-19 disease Call financed by the Carlos III Institute of Health and co-financed with ERDF funds.

Abstract

Rationale: Knowledge about the impact of the SARS-CoV-2 virus on pregnancy is still scarce and all current recommendations are based on less than 100 cases published in the literature. To identify moderate effects (such as vertical transmission, obstetric morbidity, foetal death, maternal or neonatal death) and to allow accurate risk estimates, larger sample sizes than those currently available are required.

Methods: Prospective observational study of pregnant women in whom SARS-CoV-2 infection is suspected at any time during pregnancy with positive test results for SARS-CoV-2, in order to create a registry of baseline characteristics of the pregnant woman, aspects related to the course of pregnancy and delivery, and related to the new-born, with an observation period of up to 14 days after delivery. Subsequently, several phased studies will be conducted to help establish and monitor the set of measures to improve the care of pregnant women.

Discussion: The national registry for COVID-19 in pregnancy described here is a tool for sharing and centralizing data related to exposures to SARS-CoV-2 during pregnancy in a structured way. It should speed up the process of prospectively obtaining a large unbiased data set and will collect information at national level.

Study rationale and background

On 31st December 2019, the Wuhan Municipal Health Commission (Hubei Province, China) reported on a cluster of 27 pneumonia cases of unknown aetiology, with a common exposure to a seafood and live animal wholesale market in Wuhan City, including seven serious cases (1). The first case began on 8th December 2019 with fever, dry cough, dyspnoea, and radiological findings of bilateral pulmonary infiltrates. On 7th January 2020, Chinese authorities identified a new type of virus from the Coronaviridae family as the causative agent of the outbreak, which was subsequently named SARS-CoV-2, and whose genetic sequence was shared by the Chinese authorities on 12th January. The disease caused by this new virus has been called COVID-19 by international consensus (2).

Clinical course of SARS-CoV2 infection in pregnant women

Pregnant women are known to experience immunological and physiological changes that can make them more susceptible to viral respiratory infections, including COVID-19. Several studies revealed that pregnant women with different viral respiratory diseases had a high risk of developing obstetric complications and adverse perinatal outcomes compared to non-pregnant women, due to changes in immune responses (3,4). We also know that pregnant women may be at risk of serious illness, morbidity, or mortality compared to the general population, as seen in other coronavirus infections.

Coagulation alterations have been described in people admitted to hospital for COVID-19 and, given that pregnancy confers a state of hypercoagulability, it is to be assumed that SARS-CoV2 infection in pregnant women may increase the risk of suffering from thromboembolic events, therefore, depending on the symptoms and other risk factors, they would require heparin prophylaxis (5,6). In Spain, there have been two deaths of patients with COVID-19 who had completed their pregnancies by Caesarean section, although it should be borne in mind that in both cases the Caesarean section was carried out due to severe maternal involvement (7). The causes of these deaths were related to coagulation abnormalities, in the first case due to pulmonary thromboembolism in a context of sepsis and in the second due to abruptio placentae with fulminant coagulopathy (8).

Regarding the period of gestation, no significant differences have been found between the course of the disease depending on the trimester of gestation, although most admissions for COVID-19 have occurred during the second and third trimesters (5,6,9). However, it has been shown that caesarean delivery is an independent risk factor that increases the chances of clinical worsening in pregnant women compared to vaginal delivery (7), both in asymptomatic pregnant women and in those with mild symptoms.

In China, several published studies show that in cases where the pregnant woman had pneumonia or a diagnosis of SARS-CoV-2, the delivery was often completed by caesarean section. The results of the Chinese series on 18 published cases show that 16 have resulted in caesarean section and two in vaginal deliveries. In these series, the patients showed symptoms of the disease (fever and cough) before, during and after delivery (10,11).

In the Chen H study, caesarean section was carried out between days 1 and 7 from the onset of symptoms (mean 3.3 days). One of these cases occurred after the caesarean section and in two cases a non-elective caesarean section was carried out at night (10).

In the study by Zhu H, of the 9 cases, 3 manifested in the postpartum, 2 intrapartum and 4 before delivery. In this series, two vaginal deliveries are published, with one of them being twins in a patient with symptoms (fever and cough) and the other one with symptoms starting in the postpartum period. Delivery occurred between 1 and 6 DAYS after admission (mean 3.5). Of those cases in which the symptoms occurred in the postpartum, between 1 and 3 days elapsed from delivery in this series. One case had a cough and two cases had fever (11).

Although the indications for caesarean sections are not clearly specified in the Chen H study (10), in the Zhu H study 5 were due to foetal *distress*, one case of previous uterine scar and an elective caesarean section due to maternal fever. The author suggests that maternal hypoxemia caused by infection triggers foetal hypoxia and preterm delivery. There are no data on the direct influence of COVID-19 on the intrauterine foetal status (11).

Vertical transmission

The evidence of vertical transmission (before, during or after delivery by breastfeeding) in women who acquire the infection during the third trimester of pregnancy is scarce and intrauterine transmission of the virus has not yet been totally ruled out (12).

In the Chinese series, 16 out of the 18 pregnant women gave birth by caesarean section and, although the newborns did not show SARS-CoV-2 infection, perinatal infection can have adverse effects on newborns, such as loss of foetal well-being, premature birth, respiratory distress, thrombocytopenia along with impaired liver function and even death (10,11). In Chen H's study, the amniotic fluid (only 6 samples of the 9 caesarean sections), cord and placenta analysis was negative for COVID-19, but in this series there have been no normal deliveries with prolonged dilations or passage of the foetus through the birth canal (10).

Regarding the transmission of the virus through breastfeeding, there are still contradictory data regarding the presence of the virus in breast milk. However, in theory, breastfeeding is not contraindicated in women with COVID19 (13,14).

Rationale

To date, few studies have described the impact of the SARS-CoV-2 virus on pregnancy, particularly infection occurring during the third trimester of pregnancy. The impact of the virus during the first and second trimesters of pregnancy remains unknown and must be urgently determined. All current recommendations are based on less than 50 cases published in the literature, with speculations on different aspects of management. To identify moderate effects (such as vertical transmission, foetal death, maternal or neonatal death), larger sample sizes than those currently available are required to allow accurate risk estimates. The national registry for COVID-19 in pregnancy described here is a tool for sharing and centralizing data related to exposures to SARS-CoV-2 during pregnancy in a structured way. It should speed up the process of prospectively obtaining a large unbiased data set and will collect information at national level.

Study hypothesis and objectives

Hypothesis

The COVID 19 disease affects pregnancy and has an influence on maternal and perinatal morbidity and mortality

Objectives

This study focuses on the creation of a registry of pregnant women with COVID-19 infection, which will help establish and monitor the set of measures to improve their care.

Primary objective

Collect and analyse COVID-19 cases in pregnant and postpartum women at associated health centres.

Secondary objectives

1. Establish incidence of COVID-19 infections diagnosed in pregnant women in the Spanish population.
2. Determine the variables that are associated with more maternal and neonatal morbidity.
3. Determine if there is vertical transmission.
4. Evaluate the risk of SARS-CoV-2 infection during pregnancy.
5. Characterize the clinical course of SARS-CoV-2 infection during pregnancy (for example, severity of maternal symptoms, distribution of gestational age at onset, ICU admission, mechanical ventilation, death).
6. Quantify the risk of adverse pregnancy outcomes (e.g., miscarriage, stillbirth, growth restriction) and neonatal outcomes (e.g., NICU, prematurity, death, birth defects).
7. Identify additional risk factors and risk modifiers (e.g., symptomatic infection, SARS-CoV-2 positive amniotic fluid, co-infections).

8. Develop a responsive data collection tool across a network of healthcare facilities to ensure rapid assessment of risks associated with future emerging pathogens and to help develop guidelines on this disease.
9. Strengthen capacity-building in data exchange against emerging pathogens.

The collected dataset will allow a comprehensive characterization of the risks associated with SARS-CoV-2 infection in pregnancy. In the future, the developed structure will allow a rapid assessment of the risks linked to future emerging pathogens.

Study design

Prospective observational registry study of pregnant women with suspected SARS-CoV-2 infection at any time during pregnancy with positive test results for SARS-CoV-2.

A specific database will be developed, which will include the baseline characteristics of the pregnant women, aspects related to the course of pregnancy and childbirth, and the new-born. Vertical transmission will be analysed only by means of a nasopharyngeal swab from the new-born before contact with the mother. This registry sets an observation deadline of up to 14 days after birth for the new-born and 6 weeks postpartum for the mother. The registry will be hosted on a secure https server, this server will be physically located at **OBS Covid Spain** of the company ReserArch which has a contract with the Puerta de Hierro-Majadahonda University Hospital Research Foundation.

Recruitment period will be between 1st March 2020 and 1st March 2021.

After that, different sub-analyses will be carried out with the data extracted in the registry and the secondary objectives will be addressed.

Definitions

Definition of case and healthy patient for the study

The result in the PCR test on a sample of nasopharyngeal and/or oropharyngeal swab was considered synonymous with exposure/non-exposure, defining a case as any pregnant woman with a positive result, regardless of the symptoms. Those cases with clinical presentation of COVID-19 were classified according to the WHO division for adults into: mild symptoms, mild-moderate pneumonia, severe pneumonia and septic shock (16).

The recommended samples for diagnosis are samples from the upper respiratory tract: nasopharyngeal and/or oropharyngeal swab.

A healthy patient is considered to have a negative PCR in the absence of symptoms. Patients with positive IgM or IgG serology cannot be included. The absence of serology does not prevent classifying the patient as healthy if the PCR is negative.

Definitions of symptomatic and asymptomatic patient

- Acute symptomatic patient: Positive PCR. Manifests any of the symptoms compatible with COVID-19 at the time of the study or analysis or during admission. These symptoms may vary based on new findings.
- Symptomatic patient: Patients with symptoms before 14 days of delivery, even if they no longer have symptoms at the time of delivery.
- Asymptomatic patient: Positive PCR. No symptoms compatible with COVID-19 were observed at the time of the study or analysis.
- Ex-symptomatic or cured patient: Positive or negative PCR. The patient was symptomatic (PCR+) more than 14 days before delivery and no longer has any of the symptoms compatible with COVID-19 at the time of the study or analysis. Patient can be IgM positive or negative, but shall be IgG+ if serology is available
- Symptomatic patient with sequelae: Positive or negative PCR. The patient was symptomatic (PCR+) and has any of the sequelae recognized after COVID-19 at the time of data analysis. These sequelae may vary based on new findings.

Study population and selection criteria

For the characterization of the clinical course of SARS-CoV-2 infection during pregnancy, the pre-study sample size calculation has not been carried out, as registry-based studies generally tend to be comprehensive without involving hypotheses.

Patients eligible for follow-up o target population will be any pregnant woman who is suspected or needs to be ruled out as having a SARS-CoV-2 infection at any time during pregnancy with positive test results for SARS-CoV-2 by PCR.

Information regarding each pregnant woman's demographic characteristics, comorbidities and current obstetric history was extracted from the medical history and the patient interview; subsequently, age and race were categorized according to the classification used by the Center for Disease Control and Prevention (CDC) (17).

For the selection of the control group to be collected in the same database, patients with a negative COVID-19 delivery diagnosed by PCR screening at delivery, or less than 3 days before, were considered.

Exclusion criteria

Pregnant women under the following conditions will be excluded from the registry:

- Inability to give informed consent in the absence of a legal representative.
- If, in the opinion of the researcher, findings in the physical examination, anomalies in the results of the laboratory tests or other medical, social or psychosocial factors could have a negative influence.
- Loss of follow-up data prior to 6 weeks postpartum.

Overall sample size

Currently, the percentage of pregnant patients who are being diagnosed with COVID-19 is unknown, this being one of the objectives of the study. The registry includes 100 sites that in 2019 cared for a total of 172,000 deliveries (Appendix 1 and 3).

Data sources and work plan

Patient referral sources

If the doctor in charge considers it appropriate to inform the patient about the existence and purpose of this registry, he/she may do so. At this time, consent will be requested so that the information derived from the regular monitoring of these patients is incorporated into the data registry, and that said information can be analysed and disclosed for scientific purposes in accord with the confidentiality provisions of the current legislation.

Data collection

Encrypted health-related personal data is collected prospectively using the **OBS Covid Spain** database of the company ReserArch.

The collection of data on each pregnant woman is planned from the diagnosis of a case in a pregnancy up to 6 weeks after delivery and up to 14 days postpartum in the case of the new-born, based on a manual prepared for the collection of data and for the investigator.

Hospitals enrol their pregnant patients on an ongoing basis, and the information is collected and stored anonymously in two separate phases: the first phase, during the inclusion period that occurs at the time of the SARS-CoV-2 test in the pregnant woman; the second phase, throughout the 2 weeks after birth.

For sites with existing cohorts using methods that comply with registry data collection methods (i.e., systematic inclusion of women screened for SARS-CoV-2 regardless of test results, prospective follow-up, and information

requested as shown in the variables table of appendix 5), the data collected will be entered at the time they join the registry.

Delivery monitoring: the *outcomes* include perinatal and neonatal events, which were collected at the time of delivery and the days immediately afterward. These perinatal events included gestational age, type of delivery, presence of prepartum or premature rupture of membranes before delivery, as well as any medical complications (thromboembolic events, admission to ICU, maternal death) and obstetric complications (hemorrhagic events and hypertensive disorders), while neonatal events included the 5-minute Apgar score, the pH of the umbilical artery and possible complications such as admission to the NICU and neonatal death in the follow-up period or intrauterine foetal death. The definitions of obstetric pathology follow the international criteria of the ACOG and RCOG (18-21).

Follow-up after delivery: Patient follow-up is carried out by the local investigator over the phone and the medical history is consulted from the time the patient is discharged up to 6 weeks later in order to detect late postpartum complications related to COVID 19. In the case of the new-born, the follow-up is 14 days by the investigating neonatology department by telephone and consultation of the patient's medical history to detect symptoms of COVID 19 symptoms. Complications recorded in the mother during this period (mastitis, endometritis, pulmonary embolism) as well as neonatal death or readmission of the new-born due to COVID 19 were considered outcomes of interest and analysed together with those previously described.

Data quality assurance plan

Several strategies have been implemented to reduce data errors, including a limited number of open questions, a predefined range of values where applicable, an easy-to-use case report form build that uses branching logic to reduce fields to complete, one person per site assigned to data collection, correction of systematic inconsistencies and verification of outliers. The different forms that will be used to collect the data in a systematic way are shown in appendix 1.

Heterogeneity tests

Populations from diverse genetic, socio-cultural and socio-economic backgrounds can be quite different in their characteristics. Additionally, there may be some disparities between sites with respect to the quality and quantity of data items that each site can measure. This is expected regarding a data collection that allows any site to contribute. Consequently, heterogeneity in effect estimates may be due to clinical differences between pregnant women and/or site-level differences in terms of exposure and outcome determination (reporting or assessment bias). We will formally assess heterogeneity taking into account variations in exposure, covariates, and significant outcomes, and use meta-analysis methods (Q or I2 statistics, meta-regression, and subgroup analysis) to unravel heterogeneity at central and individual levels. Briefly, we will incorporate individual-level interaction terms in one-stage analysis and random intercepts to account for central-level factors not measured in the regression models mentioned above.

EXTRACTION OF DATA FROM THE REGISTRY: SUBSTUDIES

Based on the registry prepared, data extraction will be carried out to carry out four sub-studies that address the secondary objectives.

Substudy 1: Prospective observational study on delivery and vertical transmission to the new-born.

Objective/s

1. Determine the variables that are associated with more maternal and neonatal morbidity.
2. Determine if there is vertical transmission.
3. Evaluate the risk of SARS-CoV-2 infection during pregnancy.
4. Characterize the clinical course of SARS-CoV-2 infection during delivery
5. Identify additional risk factors and risk modifiers (e.g., symptomatic infection, SARS-CoV-2 positive amniotic fluid, co-infections).

Design

Prospective observational study in 100 Spanish sites

Recruitment: 1st March 2020 to 1st March 2021. Spanish sites collected in Appendix 1.

Patients

Patients eligible for follow-up or target population will be any pregnant woman who is suspected of or needs to be ruled out as having SARS-CoV-2 infection at any time during pregnancy with positive test results for SARS-CoV-2 by PCR.

The result of the PCR test on a sample of nasopharyngeal and/or oropharyngeal swab was considered synonymous with exposure/non-exposure, defining a case as any pregnant woman with a positive result, regardless of the symptoms. Those cases with clinical presentation of COVID19 were classified according to the WHO division for adults into: mild symptoms, mild-moderate pneumonia, severe pneumonia and septic shock (16).

The recommended samples for diagnosis are samples from the upper respiratory tract: nasopharyngeal and/or oropharyngeal swab.

Primary variables

<u>Maternal characteristics</u>

**Age, mean years (range)**	
**Age groups, n (%)**	<20
	20-34
	≥35
**Time between COVID-19 diagnosis and delivery, mean days (range)**	
**Symptomatology, n (%)**	Showing COVID-19 symptoms
	Asymptomatic

<u>Delivery characteristics</u>

**Gestational age, mean weeks (range)**	
**Preterm deliveries (<37 weeks), n (%)**	
**Start of delivery, n (%)**	Spontaneous
	Induced
	Elective caesarean

**Type of delivery, n (%)**	Normal
	Instrumental
	Caesarean
**Weight at birth, mean grams (range)**	
**Neonatal tests for COVID-19, n (%)**	<12 hours from delivery (Positive ones)
	12–48 hours from delivery (Positive ones)
**Skin-to-skin within first 24 hours, n (%)**	
**Breastfeeding at immediate postpartum, n (%)**	
**Arterial pH, mean value (range)**	
**5-minute Apgar score, n (%)**	<5
	≥5
**ICU admission, n (%)**	

Evaluation 14 days after delivery, N

**Symptomatology, n (%)**	Showing COVID-19 symptoms
	Asymptomatic
**COVID-19 symptoms**	
**Mild symptoms (Symptoms reported at birth)**	Cough
	Fever
	Dyspnoea
	New olfactory or taste disorder
	Fatigue/malaise
	Altered consciousness
	Headache
	Nausea/vomiting
	Diarrhoea
**Mild-moderate pneumonia**	
**Severe pneumonia**	
**Septic shock/maternal mortality**	

The rest of the variables are defined in appendix 5

Statistics

For the statistical analysis, the SAS v9.4 software will be used.

The set of groups of variables requested and collected for each enrolled patient will be the same. The demographic and clinical characteristics of the population included will be summarized. Mean, standard deviation, minimum and maximum or median and interquartile intervals will be used for continuous variables, according to the distribution. The corresponding absolute and relative frequency will be reported for the discrete variables.

An analysis of the possible association of both the characteristics of the patients and the outcomes collected with COVID-19 infection will be carried out using Pearson's Chi-square test or Fisher's exact test and the Mann-Whitney U test (after verification of the absence of normal data by the Kolmogorov-Smirnov test). See section on Data Quality Plan and sample size. Statistical significance was established with $P < 0.05$.

Substudy 2: Prospective observational study on maternal and neonatal morbidity in pregnant women with COVID-19

Objective/s

1. Determine the variables that are associated with more maternal and neonatal morbidity.
2. Characterize the clinical course of SARS-CoV-2 infection during pregnancy (for example, severity of maternal symptoms, distribution of gestational age at onset, ICU admission, mechanical ventilation, death).
3. Identify additional risk factors and risk modifiers (e.g., symptomatic infection, SARS-CoV-2 positive amniotic fluid, co-infections).

Design

Descriptive Observational Study

Recruitment: 1st March 2020 to 1st March 2021. Spanish sites collected in Appendix 1.

Patients

Patients eligible for follow-up or target population will be any pregnant woman who is suspected of or needs to be ruled out as having SARS-CoV-2 infection at any time during pregnancy with positive test results for SARS-CoV-2 by PCR.

The result in the PCR test on a sample of nasopharyngeal and/or oropharyngeal swab was considered synonymous with exposure/non-exposure, defining a case as any pregnant woman with a positive result, regardless of the symptoms. Those cases with clinical presentation of COVID19 were classified according to the WHO division for adults into: mild symptoms, mild-moderate pneumonia, severe pneumonia and septic shock (16).

The recommended samples for diagnosis are samples from the upper respiratory tract: nasopharyngeal and/or oropharyngeal swab.

Primary variables

**Age**	Premature rupture of membranes (PRM)
**Race**	Preterm PRM
**Weight at the beginning of pregnancy**	NICU admission
**BMI: Body Mass Index**	NICU admission days
**Single or Multiple Gestation**	APGAR 5
**IVF with egg donation and IVF without egg donation**	Arterial pH
**Medical comorbidity**	Postpartum haemorrhage
**Blood group and Rh**	Abruptio placentae
**Maternal mortality**	Severe preeclampsia
**ICU admission**	HELLP
**Intrauterine foetal mortality**	Pulmonary embolism
**COVID 19 diagnosis date**	Endometritis
**Delivery < 37 weeks**	Mastitis
**Delivery Type**	

COVID-19 symptoms	
**Mild symptoms (Symptoms reported at birth)**	Cough
	Fever
	Dyspnoea
	New olfactory or taste disorder
	Fatigue/malaise
	Altered consciousness
	Headache
	Nausea/vomiting
	Diarrhoea
**Mild-moderate pneumonia**	
**Severe pneumonia**	
**Septic shock/maternal mortality**	

The rest of the variables are defined in appendix 5

Statistics

For the statistical analysis, the SAS v9.4 software will be used.

The set of groups of variables requested and collected for each enrolled patient will be the same. The demographic and clinical characteristics of the population included will be summarized. Mean, standard deviation, minimum and maximum or median and interquartile intervals will be used for continuous variables, according to the distribution. The corresponding absolute and relative frequency will be reported for the discrete variables.

An analysis of the possible association of both the characteristics of the patients and the outcomes collected with COVID-19 infection will be carried out using Pearson's Chi-square test or Fisher's exact test and the Mann-Whitney U test (after verification of the absence of normal data by the Kolmogorov-Smirnov test). See section on Data Quality Plan and sample size. Statistical significance was established with $P < 0.05$. All statistical analyses were carried out with SAS 9.4 software.

Substudy 3. Cohort study on obstetric pathology.

Objective/s

The purpose of this study was to test if pregnant patients with COVID-19 have more obstetrical morbidity than those non-infected.

- Determine the variables that are associated with more maternal and neonatal morbidity.
- Quantify the risk of adverse pregnancy outcomes (e.g., miscarriage, stillbirth, growth restriction) and neonatal outcomes (e.g., NICU, prematurity, death, birth defects).

Design

Longitudinal cohort case study to quantify the obstetrical and perinatal morbi-mortality throughout all hospitals in Spain with a universal, consecutive PCR based screening program.

Recruitment: 1st March 2020 to 1st March 2021. Spanish sites collected in Appendix 1.

Patients

Case eligibility criteria included individuals with laboratory confirmation of COVID-19 infection, irrespective of clinical signs and symptoms or the result of another serological test. Positive RNA PCR test from maternal nasopharyngeal swabs.

The selection of the control group will be collected in the same database, considering patients with negative COVID-19 delivery diagnosed by PCR screening at the time of delivery or less than 3 days before.

The delivery or deliveries that were treated in this site and that occurred within the same day, before and after the case (maximum 3 per case), are included in the control group. If the case were the last delivery of the day, the first ones of the following day could be recruited. If the case were the first of that day, the last one of the previous day could be recruited.

The data collected will be entered at the time they join the registry.

Delivery monitoring: the *outcomes* include perinatal and neonatal events, which were collected at the time of delivery and the days immediately afterward. These perinatal events included gestational age, type of delivery, presence of prepartum or premature rupture of membranes before delivery, as well as any medical complications (thromboembolic events, admission to ICU, maternal death) and obstetric complications (hemorrhagic events and hypertensive disorders), while neonatal events included the 5-minute Apgar score, the pH of the umbilical artery and possible complications such as admission to the NICU and neonatal death in the follow-up period or intrauterine foetal death. The definitions of obstetric pathology follow the international criteria of the ACOG and RCOG (18-21).

Follow-up after delivery: Patient follow-up is carried out by the local investigator over the phone and the medical history is consulted from the time the patient is discharged up to 6 weeks later in order to detect late postpartum complications related to COVID 19. In the case of the new-born, the follow-up is 14 days by the investigating neonatology department by telephone and consultation of the patient's medical history to detect symptoms of COVID 19 symptoms. Complications recorded in the mother during this period (mastitis, endometritis, pulmonary embolism) as well as neonatal death or readmission of the new-born due to COVID were considered *outcomes* of interest and analysed together with those described above.

Primary variables

Maternal characteristics

**Maternal age (years: mean/range)**	
**Age range**	18-24
	25-34
	34-49
**Ethnicity**	White European
	Latino Americans
	Black non-Hispanic
	Asian non-Hispanic
	Arab
**Nulliparous**	
**Smoking**	
**ICU admission**	
**COVID 19 diagnosis date**	
**Delivery <37 weeks**	

Maternal comorbidities

**Obesity (BMI> 30 kg/m2)**	
**Cardiovascular comorbidities**	Chronic Cardiac Disease
	Pre-pregnancy HT
**Pulmonary comorbidities**	Chronic Pulmonary Disease (not asthma)
	Asthma
**Hematologic comorbidities**	Chronic Blood Disease
	Thrombophilia
	Antiphospholipid syndrome

COVID-19 symptoms

**Mild symptoms (Symptoms reported at birth)**	Cough
	Fever
	Dyspnoea
	New olfactory or taste disorder
	Fatigue/malaise
	Altered consciousness
	Headache
	Nausea/vomiting
	Diarrhoea
**Mild-moderate pneumonia**	
**Severe pneumonia**	
**Septic shock/maternal mortality**	

Delivery Type

Premature rupture of membranes
Preterm PRM
NICU admission

NICU admission days
Postpartum haemorrhage
Abruptio placentae
Severe Preeclampsia
HELLP
Pulmonary embolism

The rest of the variables are described in appendix 5

Statistics

For the descriptive analysis, categorical variables were expressed as absolute and relative frequencies; whereas quantitative ones were expressed as mean and range (minimum-maximum values). Analysis of the possible association of both the characteristics of the patients and the *outcomes* collected with COVID-19 infection is carried out using the Pearson Chi-square test or Fisher's exact test and the Mann-Whitney U test (after checking the absence of normal data using the Kolmogorov-Smirnov test). See sections of Data Quality Plan and sample size. Statistical significance was established with $P < 0.05$. All statistical analyses were carried out with SAS 9.4 software.

For each response variable (*outcome*) a multiple logistic regression model has been carried out, refined with the backward conditional stepwise method.

The response variables are those described in the previous section. The subset of explanatory variables is different for each model and is specified in the text.

It will only be of interest the Odds Ratio (OR), its 95% CI and the significance of the “COVID” factor. The information on the rest of the explanatory variables is irrelevant since they only act as adjustment variables.

Substudy 4. Epidemiological prevalence study

Objective/s

Determine the prevalence of SARS_COV2 infection in Spanish pregnant women

Design

Cross-sectional study.

The nQuery Advisor Release 7.0 software was used to calculate the sample size, based on the available data. As we do not have data on the prevalence of COVID-19, we set an expected percentage of 50% (a situation that maximizes the sample size) of asymptomatic women during delivery. We determined the sample size for a COVID-19 delivery prevalence study with an expected prevalence of 50%, a 95% confidence level and 5% accuracy, resulting in a sample size of 1056 pregnant women.

The selection of pregnant women in the sample will follow the distribution of the delivery rate by communities and hospitals in Spain according to the INE data for 2018 (Appendix 3) (15). Subsequently, a simple random selection procedure will be carried out in each site, creating a numerical list of the women to be included in the sample (Appendix 1). Pregnant women will be assigned a consecutive number from one onwards according to their due date. Subsequently, the pregnant women who will be part of the sample will be chosen when the number that identifies them is within the table previously created through the random procedure.

Recruitment: 1st March 2020 to 1st March 2021. Spanish sites collected in the Appendix 1

Patients

Delivery of pregnant women in Spanish maternity wards with a diagnostic test for COVID-19.

Inclusion criteria:

- Single or multiple gestations of 24 weeks or more without a positive laboratory test for COVID-19 prior to delivery.
- Delivery in a public or private maternity hospital in Spain with a diagnostic test for COVID-19 using PCR Kits within 72 hours prior to delivery or 24 hours after delivery.
- The presence of comorbidity is not an exclusion criterion (asthma, diabetes mellitus, hypertension-preeclampsia, endocrine disorder, dermatological pathology or other obstetric or medical-surgical pathologies).
- Acceptance by the pregnant woman to participate in the study by means of informed consent for group study.

The result of the PCR test on a sample of nasopharyngeal and/or oropharyngeal swab was considered synonymous with exposure/non-exposure, defining a case as any pregnant woman with a positive result, regardless of the symptoms. Those cases with clinical presentation of COVID-19 were classified according to the WHO division for adults into: mild symptoms, mild-moderate pneumonia, severe pneumonia and septic shock (16).

The recommended samples for diagnosis are samples from the upper respiratory tract: nasopharyngeal and/or oropharyngeal swab.

A healthy patient is considered to have a negative PCR in the absence of symptoms. Patients with positive IgM or IgG serology cannot be included. The absence of serology does not prevent classifying the patient as healthy if the PCR is negative.

Primary variables

Age	Delivery < 37 weeks
Race	Delivery Type
Weight at the beginning of pregnancy	Premature rupture of membranes
Body Mass Index (BMI)	Preterm PRM
Blood type	NICU admission
Single or Multiple	NICU admission days
IVF	Postpartum haemorrhage
Obesity BMI> 30	Abruptio placentae
Vascular comorbidities	Severe Preeclampsia
Pulmonary comorbidity	HELLP
Maternal mortality	Pulmonary embolism
ICU admission	COVID 19 diagnosis date

The rest of the variables are described in appendix 5

Statistics

For the statistical analysis, the SAS v9.4 software will be used.

The set of groups of variables requested and collected for each enrolled patient will be the same. The demographic and clinical characteristics of the population included will be summarized. Mean, standard deviation, minimum and maximum or median and interquartile intervals will be used for continuous variables, according to the distribution. The corresponding absolute and relative frequency will be reported for the discrete variables.

The possible association of both the characteristics of the patients and the *outcomes* collected with COVID-19 infection using the Pearson Chi-square test or Fisher's exact test and the Mann-Whitney U test (after checking the absence of normal data using the Kolmogorov-Smirnov test). Statistically significant associations were considered when the p value was less than 0.05. For those categorical variables in which statistically significant differences were identified, the corresponding Odds Ratio (OR) and 95% Confidence Intervals (95% CI) were estimated using univariate logistic regression models.

Logistic regressions, both univariate and multivariate, will be performed, determining the odd ratio and its 95% confidence interval.

Management and reporting of adverse reactions

As it is an epidemiological study without a drug under study, the provisions on Pharmacovigilance in Ministerial Order SAS/3470/2009 and Royal Decree 577/2013 in relation to reporting adverse reactions to approved drugs will be followed. Investigators who are aware of possible adverse reactions during the study should notify them as quickly as possible to the Autonomous Center for Pharmacovigilance corresponding to their healthcare area, through any of the channels that the institution makes available (through the yellow card system or via online through the SINAEM website). It is also recommended that the Investigator (or reporting doctor) reports all adverse reactions to the marketing authorization holder of the drugs involved. Additionally, the conditions of use outside the marketing authorization of the drug must be reported (e.g.: off-label use, overdose, misuse, abuse and medication errors) or occupational exposure, as well as cases of suspected drug interactions, pregnancy, exposure during breastfeeding and lack of efficacy.

Ethical aspects

This study will be carried out under conditions of respect for the fundamental rights of the person and the ethical principles that affect biomedical research with human beings, following the international recommendations contained in the Declaration of Helsinki, and its subsequent revisions. Likewise, national recommendations will be followed in accordance with the Biomedical Research Law 14/2007.

During the completion of this study, the investigators will strictly adhere to the provisions of this protocol, fully completing the Case Report Form.

Informed consent

In accordance with the criteria of good clinical practice, the subjects will be duly informed of all those details concerning their participation in the study and will freely give their consent in writing or orally, recording this fact in their medical records.

Preferably written or oral consent will be sought for cases and controls chosen at random from among pregnant women who have given birth in each centre and who have COVID-19. The patient may sign the consent electronically (See appendix 7).

Safety Measures and Confidentiality

Protection of human subjects: Pregnant women and newborns participate in the registry as mentioned above. No biological samples are collected for specific analysis in this study.

The laboratory tests collected in the registry will be those carried out in each site according to its protocol. No extra lab tests will be carried out for this study and there is no handling of biological tests. The data collected through the national registry for SARS-CoV-2 in pregnancy does not contain personally identifiable information.

The data of the subjects included in it will be treated in accordance with Organic Law 3/2018, of 5th December, on the Protection of Personal Data and guarantee of digital rights.

Use of the data contained in the registry

The use of the data contained in this registry within any scientific research project will previously require:

- Approval by the local Clinical Research Ethics Committee (CREC) of the study site.
- Approval by the Scientific Committee, a body that will be constituted to give scientific output to the database.
- Signing of the data protection law contract.

A citation policy will be established for the registry, as well as for the authorship order of the different sites providing patients to each research project.

References

1. World Health Organization (WHO) [Internet]. Emergencies preparedness, response Pneumonia of Unknown Cause - China. (Date of publication: 05/01/2020). Available at: https://www.who.int/csr/don/05-january-2020-pneumonia-of-unkown-cause-china/es/

2. Health Alerts and Emergencies Coordination Center of the Ministry of Health [Internet]. Update No. 37. Coronavirus disease (COVID-19). (Update date: 03/05/2020). Available at: https://www.mscbs.gob.es/profesionales/saludPublica/ccayes/alertasActual/nCov-China/documentos/Actualizacion_37_COVID-19.pdf

3. Ministry of Health [Internet]. Management of pregnant women and newborns with COVID-19 - Technical document. 17th June 2020 version. Available at : https://www.mscbs.gob.es/profesionales/saludPublica/ccayes/alertasActual/nCov-China/documentos/Documento_manejo_embarazo_recien_nacido.pdf

4. Favre G, Pomar L, Musso D, Baud D. 2019-nCoV epidemic: what about pregnancies? Lancet 2020. 395(10224):e40. doi:10.1016/S0140-6736(20)30311-1

5. Bikdeli B, Madhavan MV, Jimenez D, et al. COVID-19 and Thrombotic or Thromboembolic Disease: Implications for Prevention, Antithrombotic Therapy, and Follow-up. J Am Coll Cardiol 2020 doi: 10.1016/j.jacc.2020.04.031 [published Online First: 15 April5.

6. Royal College of Obstetrician and Ginecologist [Internet]. Coronavirus (COVID-19) infection and pregnancy. Versión 11. Fecha de actualización: 24/07/2020. Available at: https://www.rcog.org.uk/globalassets/documents/guidelines/2020-07-24-coronavirus-covid-19-infection-in-pregnancy.pdf

7. Martínez-Perez O. , Vouga M., Cruz Melguizo S. et al. Association Between Mode of Delivery Among Pregnant Women With COVID-19 and Maternal and Neonatal Outcomes in Spain JAMA. Published online June 8, 2020. doi:10.1001/jama.2020.10125

8. Encinas B, Caño A, Marcos B, Sanz A, Rodríguez I, Hernando P et al. Spanish registry of COVID-19 screening in asymptomatic pregnant women. Spanish Journal of Epidemiology. Accepted for publication. May 2020

9. Knight M, Bunch K, Vousden N, et al. Characteristics and outcomes of pregnant women admitted to hospital with confirmed SARS-CoV-2 infection in UK: national population-based cohort study. BMJ 2020;369:m2107. doi: 10.1136/bmj.m2107

10. Chen H, Guo J, Wang C, Luo F, Yu X, Zhang W, et al. Clinical characteristics and intrauterine vertical transmission potential of COVID-19 infection in nine pregnant women: a retrospective review of medical records. Lancet 2020

11. Zhu H, Wang L, Fang C, Peng S, Zhang L, Chang G, et al. Clinical analysis of 10 neonates born to mothers with 2019-nCoV pneumonia. Transl Pediatr 2020;9:51-60.

12. 11.Baud D, Greub G, Favre G, et al. Second-Trimester Miscarriage in a Pregnant Woman With SARS-CoV-2 Infection. JAMA. 2020;323(21):2198–2200. doi:10.1001/jama.2020.7233

13. Groß R, Conzelmann C, Müller JA, Stenger S, Steinhart K, Kirchhoff F et al. Detection of SARS-CoV-2 in human breastmilk. Lancet. 2020 Jun 6;395(10239):1757-1758.

14. World Health Organization [Internet]. Clinical Management of COVID-19. (Update date: 05/27/2020). Available at: https://www.who.int/publications/i/item/clinical-management-of-severe-acute-respiratory-infection-when-novel-coronavirus-(ncov)-infection-is-suspected

15. National Institute of Statistics (INE). [Internet]. Births 2018. Births by province of residence of the mother, age of the mother and age group of the father. Visited on 05/02/2020. Available at: https://www.ine.es/jaxi/Datos.htm?path=/t20/e301/nacim/a2018/l0/&file=03001.px#!tabs-tabla

16. Worlds Health Organization. Clinical Management of COVID-19. Interim Guidance. May 27, 2020. Accessed Aug 3, 2020. Available at: https://www.who.int/publications/i/item/clinical-management-of-covid-19

17. Ellington S, Strid P, Tong VT, Woodworth K, Galang RG, Zambrano LD et al. Characteristics of Women of Reproductive Age with Laboratory-Confirmed SARS-CoV-2 Infection by Pregnancy Status. MMWR Morb Mortal Wkly Rep 2020; 69(25): 769-775.

18. American College of Obstetricians and Gynecologists' Committee on Practice Bulletins—Obstetrics. Prelabor Rupture of Membranes: ACOG Practice Bulletin, Number 217. Obstet Gynecol. 2020;135(3):e80-e97.

19. Thomson AJ; Royal College of Obstetricians and Gynaecologists. Care of Women Presenting with Suspected Preterm Prelabour Rupture of Membranes from 24+0 Weeks of Gestation: Green-top Guideline No. 73. BJOG. 2019;126(9):e152-e166.

20. Brown MA, Magee LA, Kenny LC, Karumanchi SA, McCarthy FP, Saito S et al. Hypertensive Disorders of Pregnancy: ISSHP Classification, Diagnosis, and Management Recommendations for International Practice. Hypertension. 2018;72(1):24-43.

21. Zhou F, Yu T, Du R, Fan G, Liu Y, Liu Z et al. Clinical course and risk factors for mortality of adult inpatients with COVID-19 in Wuhan, China: a retrospective cohort study. Lancet. 2020;395(10229):1054-1062.

APPENDIX 1. Registry investigators by Autonomous Community

AUTONOMOUS COMMUNITY	PROVINCE/CITY	HOSPITAL	PRINCIPAL INVESTIGATOR
ANDALUSIA	ALMERIA	**Poniente Hospital**	Marta Ruth Meca Casbas
		**Hospital Universitario Torrecárdenas**	Ana María Fernández Alonso
	CADIZ	**Jerez de la Frontera Hospital**	Susana Soldevilla Pérez
		**Puerta del Mar University Hospital Cadiz**	José Roman Broullón Molanes
		**La Línea Hospital**	Manuel Domínguez González
		**Punta Europa Hospital**	Alegria Moreno Gomez
	CORDOBA	**San Juan de Dios Hospital**	Jorge Duro Gomez
		**Reina Sofía University Hospital**	Maria Joaquina Gimeno
		**Infanta Margarita de Cabra Hospital**	Carmen Baena Luque
		**Montilla and Quirónsalud Córdoba Hospital**	Tania Manrique Gomez
	GRANADA	**Virgen de las Nieves University Hospital. Bio-health Research Institute (IBS) Granada**	Alberto Puertas Prieto
		**San Cecilio University Hospital. Bio-health Research Institute (IBS) Granada**	Africa Caño Aguilar
		**Santa Ana Hospital Motril**	Encarnacion Carmona Sánchez
	HUELVA	**Juan Ramón Jiménez Hospital**	Maria Reyes Granell Escobar
		**Riotinto Mines Hospital**	Elena Pascual Salvador
	JAEN	**Jaén Hospital Complex**	Rosario Redondo Aguilar
		**San Juan de la Cruz Hospital**	Isabel Cabello and Alba Fernández
		**Alto Guadalquivir Hospital**	Victor Muñoz Carmona
	MALAGA	**Costa del Sol Hospital**	Maria Caridad Ortiz Herrera
		**AGSE Hospital Axarquía**	M. Belén Garrido Luque
		**Virgen de la Victoria University Hospital**	Marta Camacho Caro
		**Serranía de Ronda Hospital**	Isabel Mena Piedra
		**Regional Hospital of Malaga**	Maria Suarez Arana
		**Quironsalud Malaga Hospital**	Marta García Sánchez
	SEVILLE	**Viamed Hospital, Chacón Group**	José Antonio Sainz Bueno
		**Valme Hospital**	Rosa Ostos Serna
		**Virgen del Rocío Hospital**	Lucas Cerrillos González
		**Virgen Macarena University Hospital**	Maria del Pilar Guadix Martin
ASTURIAS	ASTURIAS	**HUCA**	Jose Adánez Garcia
		**San Agustín University Hospital**	Eva Maria Oviedo Pérez
		**University Hospital of Cabueñes**	Oscar Vaquerizo Ruiz
CASTILLA LA MANCHA	CIUDAD REAL	**General University Hospital of Ciudad Real**	Antonio Sánchez Muñoz
		**La Mancha Centro General Hospital**	Mercedes Ramírez Gómez
		**University Hospital of Puerto Real**	Carmen González Macías
	CUENCA	**Virgen de la Luz Hospital**	María José Núñez Valera
CASTILLE and LEON	BURGOS	**Burgos Hospital**	Rubén Alonso Saiz
	LEON	**Leon Healthcare Complex**	Camino Fernández
	SALAMANCA	**Salamanca University Care Complex**	Ana Villalba Yarza
	VALLADOLID	**Valladolid University Clinical Hospital**	Cristina Álvarez Colomo
		**Río Hortega University Hospital**	Rut Bernardo Vega
			Alma Posadas San Juan
	ZAMORA	**Virgen de la Concha Hospital of Zamora**	Mónica Catalina Coello
CATALONIA	BARCELONA	**Hospital del Mar**	Elena Ferriols Pérez
		**Santa Creu i Sant Pau Hospital**	María Del Carmen Medina Mallén
CEUTA	CEUTA	**Hospital Universitario de Ceuta**	José Ruiz Aragon
EXTREMADURA	Cáceres	**Hospital San Pedro de Alcántara**	Juan Carlos Wizner de Alva
GALICIA	A Coruña	**Hospital Universitario A Coruña**	María Jesús Janeiro Freire
	Ferrol	**C.H, Universitario Ferrol**	Raquel Gonzalez Seoane
	Santiago	**Complejo Hospitalario de Santiago de Compostela**	Maria Begoña Dueñas Carazo
	Orense	**Complejo Hospitalario Universitario de Ourense**	Esther Alvarez Silvares
ISLAS BALEARES	Mallorca	**Hospital Son Espases**	Eva Morán Antolín
		**Hospital Comarcal de Inca**	Nofre Alomar Mateu
		**Hospital Universitario Son Llàtzer**	Albert Tubau Navarra
LA RIOJA	Logroño	**Complejo Hospitalario San Millán – San Pedro**	Ángeles Sanchez-Vegazo García
MADRID	Majadahoda	**Hospital Universitario Puerta de Hierro Majadahonda**	Begoña Encinas Pardilla
			Sara Cruz Melguizo
			Paloma Hernandez
			Amelia Sanz Lorenzana
			Rita Salvador López
	Madrid	**Hospital Infanta Sofía**	Carmen Barbancho
	Madrid	**HM Hospitales**	Pilar Monteliu
	Getafe	**Hospital Universitario de Getafe**	Pablo G Del Barrio
	Madrid	**Hospital 12 de Octubre**	Laura Forcen
			Inmaculada Mejia
			Iria Rodriguez
	Torrejon de Ardoz	**Hospital de Torrejón**	Silvia Mateos
			Irene Fernández Buhigas
	Fuenlabrada	**Hospital Universitario de Fuenlabrada**	Maria Teulon
	Aranjuez	**Hospital del Tajo**	Alejandra Maria Cano Garcia
	Pozuelo	**Hospital Quironsalud Madrid**	Olga Nieto
	Madrid	**Hospital Clínico San Carlos**	Noelia Perez Perez
	Leganes	**Hospital Universitario Severo Ochoa**	Carmen María Orizales
MURCIA	Lorca	**Hospital Rafael Méndez**	Magadalena Molina
	Murcia	**H U Virgen de la Arrixaca**	Lucia Meca
	Cartagena	**H U Santa Lucia**	Rocio Lopez Lopez
PAÍS VASCO	Alava	**Hospital Universitario Araba**	Irene Gastaca Abasolo
	Guipúzcoa	**Hospital U de Bilbao**	Mercedes Fraca Padilla
	Guipúzcoa	**Hospital de Donosti**	Jose Navarrina Martínez
	Guipúzcoa	**Hospital Goierri -Alto Urola en Zumarraga Gipuzkoa**	Carmen Álvarez
COMUNIDAD VALENCIANA	Valencia	**Hospital Universitario La Fe**	Beatriz Bueno
			Alicia Martinez
	Valencia	**Hospital Peset**	Cristina Ruiz Aguilar
	Alicante	**Hospital de Villalopo**	María Isabel Conca Rodero

APPENDIX 2: Database forms

A total of 15 forms will be completed, collected in the following table.

FORMS
Baseline form
History Form
COVID19 diagnosis
Ward/ICU admission
Pregnancy follow-up 12 weeks
Pregnancy follow-up 20 weeks
Pregnancy follow-up 35 weeks
Gestational control
Emergency visit
Delivery form
Basic new-born form
Second twin form
Lab tests
Blood type
Anaesthesia
End Form

Appendix 3: Case distribution according to Spanish provinces (INE 2018): data adapted to the study.

7		Total nacimientos	%	Total test	%
8	Total	370.827	100	1056	100
9	02 Albacete	3.040	0,82	8,66	0,82
10	03 Alicante/Alacant	14.161	3,82	40,33	3,82
11	04 Almería	7.417	2,00	21,12	2,00
12	01 Araba/Álava	2.683	0,72	7,64	0,72
13	33 Asturias	5.733	1,55	16,33	1,55
14	05 Ávila	979	0,26	2,79	0,26
15	06 Badajoz	5.210	1,40	14,84	1,40
16	07 Balears, Illes	10.285	2,77	29,29	2,77
17	08 Barcelona	46.687	12,59	132,95	12,59
18	48 Bizkaia	7.945	2,14	22,62	2,14
19	09 Burgos	2.351	0,63	6,69	0,63
20	10 Cáceres	2.611	0,70	7,44	0,70
21	11 Cádiz	10.246	2,76	29,18	2,76
22	39 Cantabria	3.805	1,03	10,84	1,03
23	12 Castellón/Castelló	4.580	1,24	13,04	1,24
24	13 Ciudad Real	3.676	0,99	10,47	0,99
25	14 Córdoba	6.209	1,67	17,68	1,67
26	15 Coruña, A	7.132	1,92	20,31	1,92
27	16 Cuenca	1.303	0,35	3,71	0,35
28	20 Gipuzkoa	5.472	1,48	15,58	1,48
29	17 Girona	6.745	1,82	19,21	1,82
30	18 Granada	7.441	2,01	21,19	2,01
31	19 Guadalajara	2.114	0,57	6,02	0,57
32	21 Huelva	4.256	1,15	12,12	1,15
33	22 Huesca	1.651	0,45	4,70	0,45
34	23 Jaén	4.886	1,32	13,91	1,32
35	24 León	2.633	0,71	7,50	0,71
36	25 Lleida	3.497	0,94	9,96	0,94
37	27 Lugo	1.959	0,53	5,58	0,53
38	28 Madrid	57.554	15,52	163,90	15,52
39	29 Málaga	13.480	3,64	38,39	3,64
40	30 Murcia	14.675	3,96	41,79	3,96
41	31 Navarra	5.398	1,46	15,37	1,46
42	32 Ourense	1.486	0,40	4,23	0,40
43	34 Palencia	983	0,27	2,80	0,27
44	35 Palmas, Las	7.742	2,09	22,05	2,09
45	36 Pontevedra	5.983	1,61	17,04	1,61
46	26 Rioja, La	2.330	0,63	6,64	0,63
47	37 Salamanca	2.075	0,56	5,91	0,56
48	38 Santa Cruz de Tenerife	7.033	1,90	20,03	1,90
49	40 Segovia	1.058	0,29	3,01	0,29
50	41 Sevilla	17.094	4,61	48,68	4,61
51	42 Soria	606	0,16	1,73	0,16
52	43 Tarragona	6.637	1,79	18,90	1,79
53	44 Teruel	947	0,26	2,70	0,26
54	45 Toledo	5.712	1,54	16,27	1,54
55	46 Valencia/València	19.277	5,20	54,89	5,20
56	47 Valladolid	3.463	0,93	9,86	0,93
57	49 Zamora	896	0,24	2,55	0,24
58	50 Zaragoza	7.379	1,99	21,01	1,99
59	51 Ceuta	972	0,26	2,77	0,26
60	52 Melilla	1.340	0,36	3,82	0,36

APPENDIX 4: List of sites

No.	Name of the site	No.	Name of the site
1	Puerta de Hierro Majadahonda University Hospital	51	Axarquia AGSE Hospital
2	Valladolid University Clinical Hospital	52	HUCA
3	Virgen de la Arrixaca University Hospital - Murcia	53	Virgen de la Victoria University Hospital
4	Getafe University Hospital	54	Santa Ana Hospital Motril
5	Araba University Hospital- Àlava	55	Santa Creu i Sant Pau Hospital
6	Donostia Hospital	56	FHSJD of Martorell
7	Viamed Hospital, Chacon Group	57	Bilbao University Hospital
8	Poniente Hospital	58	QuironSalud Marbella Hospital
9	Costa del Sol Hospital	59	HM Hospitals
10	San Millán Hospital Complex - San Pedro de la Rioja	60	Dexeus University Hospital
11	Severo Ochoa Leganes University Hospital	61	Virgen del Castillo Hospital
12	University Hospital Complex of Ourense	62	Ceuta University Hospital
13	Quironsalud Clideba Hospital	63	Quironsalud Murcia Hospital
14	Rio Hortega University Hospital	64	Northwest Regional Hospital
15	Virgen Concha Hospital - Zamora	65	Parc Taulí University Hospital - Sabadell
16	Jerez de la Frontera Hospital	66	Infanta Margarita de Cabra Hospital
17	Santiago de Compostela Hospital Complex	67	San Agustín University Hospital
18	Valme Hospital	68	University Hospital of Cabueñes
19	October 12 Hospital	69	Arnau de Vilanova University Hospital
20	Torrejón Hospital	70	Montilla and Quironsalud Cordoba Hospital
21	Los Arcos del Mar Menor University Hospital	71	Infanta Sofía Hospital
22	Infanta Leonor University Hospital	72	Serranía de Ronda Hospital
23	Lucus Augusti Hospital	73	San Juan de la Cruz Hospital
24	General University Hospital of Ciudad Real	74	Tajo Hospital
25	A Coruña University Hospital Complex	75	La Linea Hospital
26	Rafael Méndez Hospital	76	Salamanca University Care Complex
27	University Hospital of Fuenlabrada	77	Quironsalud Madrid Hospital
28	La Mancha Centro General Hospital	78	San Carlos Clinical Hospital
29	University Hospital Complex of Pontevedra	79	Alto Guadalquivir Hospital, Andújar
30	Leon Healthcare Complex	80	Mendaro Hospital
31	Ferrol University Hospital	81	University Clinic of Navarra
32	Gregorio Marañón University General Hospital	82	Dr Peset Valencia Hospital

33	San Juan de Dios Hospital	83	Goierri Hospital -Alto Urola - Zumarraga Gipuzkoa
34	Alvaro Cunqueiro Hospital	84	Sant Joan de Reus Hospital
35	Reina Sofía University Hospital	85	General Hospital of L'Hospitalet
36	Virgen de las Nieves University Hospital. Bio-health Research Institute (IBS) Granada	86	La Fe University Hospital
37	Puerta del Mar University Hospital Cadiz	87	Burgos Hospital
38	General University Hospital Santa Lucía de Cartagena	88	Torrevieja Hospital
39	Torrecardenas University Hospital	89	Santa Caterina Hospital
40	University Hospital of Puerto Real	90	Josep Trueta Girona University Hospital
41	Juan Ramón Jiménez Hospital	91	Regional Hospital of Malaga
42	Virgen del Rocío Hospital	92	Inca Regional Hospital
43	Hospital del Mar	93	Germans Trias University Hospital
44	Son Espases Hospital	94	Virgen de la Luz Hospital
45	Virgen Macarena University Hospital	95	Quironsalud Malaga Hospital
46	San Cecilio University Hospital. Bio-health Research Institute (IBS) Granada	96	Riotinto Mines Hospital
47	San Pedro de Alcántara Hospital	97	Son Llàtzer University Hospital - Palma de Mallorca
48	Jaen Hospital Complex	98	Joan XXIII University Hospital - Tarragona
49	Hospital da Barbanza	99	La Paz Hospital
50	La Inmaculada Hospital	100	Vinalopo Hospital
		101	Punta Europa Hospital

APPENDIX 5. List of variables (separate Excel document)
