# Supplementary material for: The association between SARS-CoV-2 infection and preterm delivery: a prospective study with a multivariable analysis
Source: BMC Pregnancy Childbirth. 2021 Apr 1;21:273. doi: 10.1186/s12884-021-03742-4 (PMC8016158; doi:10.1186/s12884-021-03742-4)
Supplement: Supplementary file 2 — Additional file 2: Table S1. List of hospitals included in the study (n = 45); and Table S2. STROBE Statement—checklist of items that should be included in reports of observational studies. [file 12884_2021_3742_MOESM2_ESM.docx]

**Supplementary Table 1.** List of hospitals included in the study (n=45).

| **HOSPITAL** |
| --- |
| Hospital Universitari Dexeus - Grupo Quirónsalud |
| Hospital Universitario La Paz |
| Hospital Universitario Puerta de Hierro |
| Hospital Clínico San Carlos |
| Hospital de Santa Caterina |
| Hospital Universitario de Salamanca |
| Hospital Universitario Quirónsalud Pozuelo de Alarcón |
| Hospital Univesitario de Getafe |
| Hospital Doce de Octubre |
| Hospital Univesitario Severo Ochoa |
| Hospital La Fe |
| Hospital Sant Joan de Reus |
| Hospital Universitario de Ferrol |
| Hospital Universitario de Girona Doctor Josep Trueta |
| Complejo Hospitalario A Coruña |
| HM Hospitales |
| Hospital de Torrejón |
| Hospital d'Inca |
| Hospital Parc Taulí |
| Hospital San Pedro Alcántara |
| Hospital Universitario Río Hortega |
| Hospital Arnau de Vilanova |
| Hospital Clínico Universitario de Valladolid |
| Hospital General Universitario de Ciudad Real |
| Hospital Universitari Germans Trias i Pujol |
| Hospital Universitario de Burgos |
| Hospital Universitario de Tarragona Juan XXIII |
| Hospital Universitario Infanta Sofía |
| Hospital de Poniente |
| Hospital Universitario de Cabueñes |
| Hospital Universitario Son Espases |
| Hospital Virgen de la Luz |
| Complejo Asistencial de León |
| Hospital Universitario Son Llàtzer |
| Hospital del Tajo |
| Hospital Jerez de la Frontera |
| Hospital Universitario Araba-Txagorritxu |
| Hospital Universitario Central de Asturias |
| Hospital Universitario Virgen de las Nieves |
| Hospital Universitario Virgen de Valme |
| Hospital Universitario Virgen Macarena |
| Hospital Virgen de la Concha |
| Hospital Regional Universitario de Málaga |
| Hospital Infanta Margarita |
| Hospital Universitario de Fuenlabrada |

**Supplementary Table 2.** STROBE Statement—checklist of items that should be included in reports of observational studies.

|  | **Item No.** | **Recommendation** | **Page  No.** | **Relevant text from manuscript** |
| --- | --- | --- | --- | --- |
| **Title and abstract** | 1 | (*a*) Indicate the study’s design with a commonly used term in the title or the abstract | 1 and 7 |  |
|  |  | (*b*) Provide in the abstract an informative and balanced summary of what was done and what was found | 7 |  |
| **Introduction** | | | |  |
| Background/rationale | 2 | Explain the scientific background and rationale for the investigation being reported | 9 |  |
| Objectives | 3 | State specific objectives, including any prespecified hypotheses | 9 |  |
| **Methods** | | | |  |
| Study design | 4 | Present key elements of study design early in the paper | 10-11 |  |
| Setting | 5 | Describe the setting, locations, and relevant dates, including periods of recruitment, exposure, follow-up, and data collection | 10-12, Figure 1 and Table S1 |  |
| Participants | 6 | (*a*) *Cohort study*—Give the eligibility criteria, and the sources and methods of selection of participants. Describe methods of follow-up  *Case-control study*—Give the eligibility criteria, and the sources and methods of case ascertainment and control selection. Give the rationale for the choice of cases and controls  *Cross-sectional study*—Give the eligibility criteria, and the sources and methods of selection of participants | 10-12 and Figure 1 |  |
|  |  | (*b*) *Cohort study*—For matched studies, give matching criteria and number of exposed and unexposed  *Case-control study*—For matched studies, give matching criteria and the number of controls per case | 10-11 and Figure 1 |  |
| Variables | 7 | Clearly define all outcomes, exposures, predictors, potential confounders, and effect modifiers. Give diagnostic criteria, if applicable | 10-13 and registry protocol: ClinicalTrials.gov, NCT04558996 |  |
| Data sources/ measurement | 8* | For each variable of interest, give sources of data and details of methods of assessment (measurement). Describe comparability of assessment methods if there is more than one group | 12 and registry protocol: ClinicalTrials.gov, NCT04558996 |  |
| Bias | 9 | Describe any efforts to address potential sources of bias | 10-13 |  |
| Study size | 10 | Explain how the study size was arrived at | 10-11 and Figure 1 |  |

Continued on next page

| Quantitative variables | 11 | Explain how quantitative variables were handled in the analyses. If applicable, describe which groupings were chosen and why | 12 and registry protocol: ClinicalTrials.gov, NCT04558996 |  |
| --- | --- | --- | --- | --- |
| Statistical methods | 12 | (*a*) Describe all statistical methods, including those used to control for confounding | 12-14 and registry protocol: ClinicalTrials.gov, NCT04558996 |  |
|  |  | (*b*) Describe any methods used to examine subgroups and interactions | No subgroup nor interaction analysis was carried out because absence of scientifically sound interactions among the variables of interest was assumed based on previous findings/knowledge |  |
|  |  | (*c*) Explain how missing data were addressed | 12-14 |  |
|  |  | (*d*) *Cohort study*—If applicable, explain how loss to follow-up was addressed  *Case-control study*—If applicable, explain how matching of cases and controls was addressed  *Cross-sectional study*—If applicable, describe analytical methods taking account of sampling strategy | 12 and registry protocol: ClinicalTrials.gov, NCT04558996 |  |
|  |  | (*e*) Describe any sensitivity analyses | No sensitivity analysis was carried out |  |
| **Results** | | | | |
| Participants | 13* | (a) Report numbers of individuals at each stage of study—eg numbers potentially eligible, examined for eligibility, confirmed eligible, included in the study, completing follow-up, and analysed | Figure 1 |  |
|  |  | (b) Give reasons for non-participation at each stage | 10-12 and Figure 1 | Dropout cases: incomplete information in the registry database, did not participate in the six-week postpartum follow-up and/or voluntary withdrawal of the patient. Patient or neonate condition could not be assessed by phone after delivery |
|  |  | (c) Consider use of a flow diagram | Figure 1 |  |
| Descriptive data | 14* | (a) Give characteristics of study participants (eg demographic, clinical, social) and information on exposures and potential confounders | 14-15 and Table 1 |  |
|  |  | (b) Indicate number of participants with missing data for each variable of interest | Tables 1-2 |  |
|  |  | (c) *Cohort study*—Summarise follow-up time (eg, average and total amount) | 12 |  |
| Outcome data | 15* | *Cohort study*—Report numbers of outcome events or summary measures over time | 15-16 and Table 2 |  |
|  |  | *Case-control study—*Report numbers in each exposure category, or summary measures of exposure |  |  |
|  |  | *Cross-sectional study—*Report numbers of outcome events or summary measures |  |  |
| Main results | 16 | (*a*) Give unadjusted estimates and, if applicable, confounder-adjusted estimates and their precision (eg, 95% confidence interval). Make clear which confounders were adjusted for and why they were included | 13, 15-16 and Table 3 |  |
|  |  | (*b*) Report category boundaries when continuous variables were categorized | Table 1 |  |
|  |  | (*c*) If relevant, consider translating estimates of relative risk into absolute risk for a meaningful time period |  |  |

Continued on next page

| Other analyses | 17 | Report other analyses done—eg analyses of subgroups and interactions, and sensitivity analyses | No subgroup, interaction nor sensitivity analysis was carried out |  |
| --- | --- | --- | --- | --- |
| **Discussion** | | | | |
| Key results | 18 | Summarise key results with reference to study objectives | 16 |  |
| Limitations | 19 | Discuss limitations of the study, taking into account sources of potential bias or imprecision. Discuss both direction and magnitude of any potential bias | 17-18 |  |
| Interpretation | 20 | Give a cautious overall interpretation of results considering objectives, limitations, multiplicity of analyses, results from similar studies, and other relevant evidence | 18-20 |  |
| Generalisability | 21 | Discuss the generalisability (external validity) of the study results | 17-20 |  |
| **Other information** | |  | | |
| Funding | 22 | Give the source of funding and the role of the funders for the present study and, if applicable, for the original study on which the present article is based | 21 |  |

*Give information separately for cases and controls in case-control studies and, if applicable, for exposed and unexposed groups in cohort and cross-sectional studies.

**Note:** An Explanation and Elaboration article discusses each checklist item and gives methodological background and published examples of transparent reporting. The STROBE checklist is best used in conjunction with this article (freely available on the Web sites of PLoS Medicine at http://www.plosmedicine.org/, Annals of Internal Medicine at http://www.annals.org/, and Epidemiology at http://www.epidem.com/). Information on the STROBE Initiative is available at www.strobe-statement.org.
