# Supplementary material for: The association between SARS-CoV-2 infection and preterm delivery: a prospective study with a multivariable analysis
Source: BMC Pregnancy Childbirth. 2021 Apr 1;21:273. doi: 10.1186/s12884-021-03742-4 (PMC8016158; doi:10.1186/s12884-021-03742-4)
Supplement: Supplementary file 3 — Additional file 3. Patient Consent Form. [file 12884_2021_3742_MOESM3_ESM.pdf]

Additional File 3. Patient Consent Form

COVID-19 PANDEMIC PREGNANCY REGISTRY

INTRODUCTION:

We are writing to inform you about a research study in which you are invited to participate: a study of the influence of SARS-CoV-2 on pregnancy, by comparing pregnant women with SARS-CoV-2 to pregnant women without. The project has a favorable report and approval from a local Drug Research Ethics Committee.

Our intention is that you receive correct and sufficient information so that you can evaluate and judge whether or not you want to participate in this study. To do this, please read this information sheet carefully and we will clarify any doubts you may have. In addition, you can consult with the people you consider appropriate.

VOLUNTARY PARTICIPATION:

You should know that your participation is voluntary and that you can decide not to participate. If you decide to participate, you can change your decision and withdraw your consent at any time, without altering your relationship with your doctor or harming your health care.

OBJECTIVE OF THE STUDY:

The main objective of this study is the creation of a registry of PREGNANT WOMEN with SARS-CoV-2 infection that will help to establish and monitor the package of measures to improve their care.

In addition to the creation of a registry of SARS-CoV-2 infected PREGNANT WOMEN, your participation will help to establish and monitor the package of measures to improve their care and conduct studies on the risk factors of patients and the evolution of the newborn, for future second waves and outbreaks of the pandemic.

STUDY PROCEDURES:

If you agree to participate in the registry you will not have to do anything else, we will simply record some data about you, your baby and your delivery and use the tests that are normally performed on all mothers and newborns. No tests or other biological samples (placenta, umbilical cord for example) will be collected specifically for this study and no biological material will be stored.

RISKS AND INCONVENIENCES DERIVED FROM YOUR PARTICIPATION IN THE STUDY:

There are no risks from participation since no additional tests will be performed, only data from your medical history is collected. The only risk would be that related to the handling of personal information, but, as we will explain later, strict measures have been established to preserve your confidentiality.

POTENTIAL BENEFITS:

No direct benefit is expected from your participation in the study. However, the knowledge gained from this study can help medical progress and therefore help others.

You will not receive any economic benefit from the transfer of the data provided, nor will you have any rights to possible commercial benefits from the discoveries that may arise as a result of the research carried out.

RIGHT TO REVOKE CONSENT:

If you change your mind regarding your participation in the study and the transfer of the data provided, you have the right to revoke it through the principal investigator of the study. However, you should know that the results obtained may be kept in compliance with the corresponding legal obligations.

FINANCIAL COMPENSATION:

There is no financial compensation for participation, even for doctors and midwives. This study is partially funded by the *Institute of Health Carlos III* for the recruitment of research staff, building of professional databases, statistical analysis, management and dissemination of knowledge.

PROTECTION OF PERSONAL DATA:

The promoter and the center are committed to the fulfillment of the Regulation 2016/679 of April the 27th regarding the protection of individuals in relation to the processing of their personal data (hereinafter the "Regulation") and the Spanish regulations applicable (Organic Law 3/2018 of December the 5th, Protection of Personal Data and guarantee of digital rights).

The data collected for the study will be identified by a code, so that it does not include information that can identify you directly. Access to your personally identifiable information will be restricted to the study physician/collaborators, health authorities, the Research Ethics Committee and personnel authorized by the sponsor, when necessary to verify the data, study procedures, and compliance with standards of good clinical practice; but always maintaining confidentiality. Your identity may be disclosed in exceptional cases, such as medical emergencies or legal requirements. The treatment, communication and transfer of personal data of all participants will be in accordance with applicable regulations.

All the information we ask you for is necessary to participate in this trial and it is mandatory to provide it in order to guarantee the correct development of the trial. The data collected for the study will be kept 25 years after its completion. After that, your personal information will only be kept by the center for your health care.

According to the current regulations, you have rights over your personal data. However, we inform you that there are some limitations in order to guarantee the validity of the investigation and to comply with the legal duties.

To exercise your rights, please contact the study's principal investigator. Likewise, we inform you of your right to file a complaint to the Data Protection Agency in the case there is any action carried by the Promoter or the Center that you consider to violate your rights.

Your encrypted data may be transmitted to third parties and to other countries always for the same purposes of the described study or for its use in scientific publications and always maintaining the confidentiality of the same according to the legislation in force (in no case will they contain information that can identify you directly, such as name and surname, initials, address, social security number, etc.).

Both the Center and the main researcher are responsible for the processing of your data and are committed to comply with data protection regulations.

CONTACT:

If during your participation you have any questions or need more information, please contact OSCARMARTINEZGINE@GMAIL.COM

*Fields required

E-mail address*: ___________________________________

NEXT (click here to continue)

---Next page--

Name*: _______________________

First Surname*: ________________________

Second Surname: _____________________________

ID or Passport or Resident' card*: ____________________________

Hospital where you were/will be delivered: __________________________________

NEXT (click here to continue)

---Next page--

Answer the following:

- ○ I have read the information sheet I was given about the study.
- ○ I have been able to ask questions about the study by email
- ○ I have received enough information about the study.
- ○ I understand that my participation is voluntary.
- ○ I understand that I may withdraw from the study

I GIVE MY CONSENT:

- I freely agree to participate in this study and consent to the access and use of my data under the conditions detailed in the information sheet.

<u>SEND</u> (click here to finish)

--End---
